# Jellyfish detritus supports niche partitioning and metabolic interactions among pelagic marine bacteria

**DOI:** 10.1186/s40168-023-01598-8

**Published:** 2023-07-21

**Authors:** Tinkara Tinta, Zihao Zhao, Barbara Bayer, Gerhard J. Herndl

**Affiliations:** 1 Marine Biology Station Piran, National Institute of Biology, Piran, Slovenia; 2 Department of Functional and Evolutionary Ecology, Bio-Oceanography and Marine Biology Unit, University of Vienna, Vienna, Austria; 3 Department of Microbiology and Ecosystem Science, Centre for Microbiology and Environmental Systems Science, University of Vienna, Vienna, Austria; 4 NIOZ, Department of Marine Microbiology and Biogeochemistry, Royal Netherlands Institute for Sea Research, Utrecht University, Den Burg, The Netherlands; 5 Vienna Metabolomics & Proteomics Center, University of Vienna, Vienna, Austria

**Keywords:** Jellyfish detritus, Microbial consortia, Metagenomics, Metaproteomics, Exoproteomics

## Abstract

**Background:**

Jellyfish blooms represent a significant but largely overlooked source of labile organic matter (jelly-OM) in the ocean, characterized by a high protein content. Decaying jellyfish are important carriers for carbon export to the ocean’s interior. To accurately incorporate them into biogeochemical models, the interactions between microbes and jelly-OM have yet to be fully characterized. We conducted jelly-OM enrichment experiments in microcosms to simulate the scenario experienced by the coastal pelagic microbiome after the decay of a jellyfish bloom. We combined metagenomics, endo- and exo-metaproteomic approaches to obtain a mechanistic understanding on the metabolic network operated by the jelly-OM degrading bacterial consortium.

**Results:**

Our analysis revealed that OM released during the decay of jellyfish blooms triggers a rapid shuffling of the taxonomic and functional profile of the pelagic bacterial community, resulting in a significant enrichment of protein/amino acid catabolism-related enzymes in the jelly-OM degrading community dominated by *Pseudoalteromonadaceae*, *Alteromonadaceae* and *Vibrionaceae*, compared to unamended control treatments. In accordance with the proteinaceous character of jelly-OM, *Pseudoalteromonadaceae* synthesized and excreted enzymes associated with proteolysis, while *Alteromonadaceae* contributed to extracellular hydrolysis of complex carbohydrates and organophosphorus compounds. In contrast, *Vibrionaceae* synthesized transporter proteins for peptides, amino acids and carbohydrates, exhibiting a cheater-type lifestyle, i.e. benefiting from public goods released by others. In the late stage of jelly-OM degradation, *Rhodobacteraceae* and *Alteromonadaceae* became dominant, growing on jelly-OM left-overs or bacterial debris, potentially contributing to the accumulation of dissolved organic nitrogen compounds and inorganic nutrients, following the decay of jellyfish blooms.

**Conclusions:**

Our findings indicate that specific chemical and metabolic fingerprints associated with decaying jellyfish blooms are substantially different to those previously associated with decaying phytoplankton blooms, potentially altering the functioning and biogeochemistry of marine systems. We show that decaying jellyfish blooms are associated with the enrichment in extracellular collagenolytic bacterial proteases, which could act as virulence factors in human and marine organisms’ disease, with possible implications for marine ecosystem services. Our study also provides novel insights into niche partitioning and metabolic interactions among key jelly-OM degraders operating a complex metabolic network in a temporal cascade of biochemical reactions to degrade pulses of jellyfish-bloom-specific compounds in the water column.

Video Abstract

**Supplementary Information:**

The online version contains supplementary material available at 10.1186/s40168-023-01598-8.

## Introduction

Nuisance blooms of specific organisms are increasing in the world’s oceans, linked to natural oscillations of populations, anthropogenic impacts, climate change and associated deterioration of marine ecosystems ([1–4] and references therein). Gelatinous zooplankton or jellyfish (hereinafter the cnidarian subphylum Medusozoans) are one of the most remarkable examples of bloom-forming organisms in the ocean and are potentially on the rise [5–8]. The global mean jellyfish standing stock was recently estimated to amount to ~ 510 Tg C in the epipelagic ocean [9], representing ~ 14% of the global phytoplankton biomass [10] and ~ 20% of global zooplankton biomass [11]. Jellyfish blooms often occur seasonally and are short-lived (weeks to months), after which they abruptly collapse en masse, exhibiting dynamics described as boom-and-bust, and induce major perturbation to the marine ecosystem [12–14].

As the bloom decays, the large influx of detrital matter causes a distortion to the ambient seawater organic matter pool by releasing bloom-specific compounds, which are degraded at different rates in a cascade by specific members of the marine food web [15–19]. During a typical bloom event, with 10 jellyfish individuals per m^3^, about 100 mg of jellyfish detritus per litre becomes suddenly available to other organisms [20]. Even though jellyfish and their carcasses are grazed and scavenged to some extend [21], the amount of released organic matter (OM) in a short period of time is too large to be solely top-down controlled and sometimes intact jellyfish corpse continue sinking through the water column as jelly-falls [22, 23]. In this context, jellyfish were recently recognized as important agents of carbon export to the ocean’s interior [24, 25] potentially contributing 32–40% to the total global particulate organic carbon export [9]. However, it has been recently estimated that about half of the OM stored in the jellyfish biomass is present in the form of dissolved organic matter (DOM), which is rapidly leaching into the ambient seawater being readily and exclusively accessible to marine microbes propelling biogeochemical cycles at the base of the oceanic food web [20, 26, 27]. This implies that pelagic microbes play a significant role in determining the fate of detrital jelly-OM, with important implications for the cycling of jelly-OM in the ocean. Potential routes of jellyfish detritus in the ocean were recently revised and the importance of the largely overlooked processing of jelly-OM via the microbial loop was highlighted [28, 29].

What makes jellyfish blooms so different from phytoplankton blooms? Gelatinous organisms are composed to > 98% of water, but their organic part is rich in proteins (72 ± 14% of total OM, [30, 31], as reflected in the low C:N ratio of their biomass (~ 4.6 ± 0.1:1; [32])), easily accessible (e.g. lack of chitinous exoskeleton) and labile as in less than 2 days the majority of jelly-DOM can be degraded by coastal microbial assemblage in an enclosed system [29]. We showed that the decay of a typical coastal bloom of the cosmopolitan bloom-forming scyphozoan jellyfish *Aurelia aurita* s.l. represents an enrichment of ∼44 µmol L^−1^ of dissolved organic carbon (DOC), ∼13 µmol L^−1^ of total dissolved nitrogen (TDN) (mostly dissolved organic nitrogen (DON) compounds), ∼11 µmol L^−1^ of total hydrolysable dissolved amino acids (THDAA) (∼55% as dissolved free amino acids (DFAA) with a considerable amount of free glycine and taurine) and a substantial amount of orthophosphate (PO_4_^3−^) (∼0.6 µmol L^−1^) [20]. The large amount of labile, high-quality proteinaceous DOM released during jellyfish bloom decay can cause agitation to the ambient DOM pool, which is otherwise mostly fuelled by phytoplankton-derived complex high molecular weight (HMW) organic matter compounds ([33–37] and references therein). Studies have shown that jelly-OM supports rapid growth of bacteria (specific growth rates in the range between 0.2 d^−1^ to 7 d^−1^ depending on the jellyfish species, environment and ecosystem characteristics, [29]), which is on average higher than usually reported for the ocean’s microbiota (0.1–1 d^−1^, [38]) or bacteria growing on phytoplankton-derived OM (up to 2.2 d^−1^, [15]). Pelagic bacteria growing on jelly-OM exhibit high growth efficiencies (65 ± 27%, [20]) as compared to bacteria growing on phytoplankton detritus (~ 20%, [39]). This is important in the light of predictions that ocean microbes could be facing an increase of gelatinous compounds within the DOM pool in the future [40]. Taxonomic profiling of jelly-OM degrading consortia highlighted the key role of *Pseudoalteromonas*, *Vibrio* and *Alteromonas*, accounting for > 90% of all metabolically active jelly-OM degraders [20, 41–43], in contrast to bacterial populations following the decay of phytoplankton blooms [17]. Microbial processing of jelly-OM resulted in the accumulation of specific dissolved free and combined amino acids and inorganic nutrients [20, 29]. To accurately integrate jellyfish blooms into oceanic biogeochemical cycles, we need to better understand the interactions between jelly-OM and microbes.

Recent methodological advances in analytical chemistry, metagenomics and metaproteomics allow us to link individual compounds within the complexity of the DOM pool to microbial metabolism, providing direct evidence of physiological and metabolic activities in microbial communities under particular environmental conditions [44–49]. Here, we merged the power of metagenomics and metaproteomics approaches to provide first insights into the metabolic network operated by jelly-OM degrading microbial consortia in the pelagial.

## Materials and methods

### Experimental design

A detailed description of the experimental design is provided in Tinta et al. [20]. Briefly, we conducted two consecutive short-term microcosm experiments to simulate the scenario potentially experienced by coastal pelagic microbial communities after the decay of a bloom of the cosmopolitan *Aurelia aurita* s.l., which forms large aggregations in several coastal ecosystems worldwide, with reported higher frequency over last decades. The rationale behind conducting experiments in a confined system, like microcosms, rather than in situ was twofold: (i) it allowed us to follow the dynamics of the microbial community in time using metagenomic and metaproteomic approaches in biological triplicates *vs* control treatments, (ii) due to the patchiness of jellyfish blooms and our current inability to track jellyfish populations remotely in situ experiments are challenging given the changing environmental conditions over days to week in coastal seas. Nevertheless, we tried to mimic environmental conditions as much as possible. For each experiment we filled six borosilicate glass flasks with 0.2 µm filtered aged seawater (ASW), which was inoculated with a 1.2-µm prefiltered prokaryotic coastal community collected in near-surface waters of the northern Adriatic Sea in a ratio of ASW:bacterial inoculum of 9:1. The final volume in the experimental bottles was 5 and 10 L in Experiment I and II, respectively. For each experiment, three of the experimental bottles received 100 mg of jellyfish dry matter L^−1^, representing the jelly-OM treatment (J1, J2, J3) to mimic typical bloom conditions in a coastal ecosystem (i.e. during a typical *A. aurita* bloom in the Adriatic Sea there were on average at least 10 jellyfish per m^3^ near the surface of the water column, each having a dry mass of ~ 10 g, which equals to 100 g jelly-DM m^−3^ [20]). Three experimental bottles remained unamended with jelly-OM and served as control (C1, C2, C3). All bottles were incubated in the dark at in situ temperature (~ 24 °C) and mixed gently prior to subsampling.

The rationale behind using jellyfish dry matter over fresh tissue was twofold: (i) it allowed us to obtain a more homogenous distribution of jelly-OM in the experimental flasks and hence a better reproducibility of biological replicates and (ii) freeze-drying of jellyfish tissue as part of pre-processing described in detail in Tinta et al. [20], preserved better the biochemical properties of labile jelly-OM. At the same time, any potential microbiota associated with jellyfish most likely did not survive the pre-processing (i.e. jellyfish tissue was stored at − 20 °C immediately after collection, then freeze-dried at − 45 °C for 7 days and then grinded with pestle and mortal and stored in acid-cleaned pre-combusted glass vials at − 20 °C until used in the experiments). The absence of microbiota in dry jellyfish matter was also confirmed using an epifluorescence microscope as part of jellyfish leaching experiments, described in Tinta et al. [20]. The fact that we removed jellyfish-associated microbiome was in line with the main aim of our study, which was to understand the degradation of jellyfish detritus by ambient pelagic marine microbial assemblage.

However, we do acknowledge that the pre-processing of jelly-OM we applied increased the surface area of jelly-OM particles and in this way potentially affected jelly-OM-microbe interactions, most probably by making certain compounds more accessible to the microbiota. However, we also showed in our previous work [20] that about half of the jelly-OM is composed of dissolved organic matter, which is readily and exclusively accessible to microbes and this part is not affected by our pre-processing. In both experiments we followed the quantity and quality of individual compounds of the DOM pool and the composition and metabolic profile of the microbial community. In Experiment I, we took subsamples at 0, 6, 12, and 24 h and terminated the experiment after 32 h, when the bacterial community reached its late exponential growth phase. In Experiment II, we subsampled the flasks at 0, 12, 32, 46, 56, and 80 h and terminated the experiment after 84 h, when the bacterial community entered its decay phase. For each subsampling, about 250 mL was removed from the flasks, leaving ca. 2/3 of the initial volume in all the flasks at the end of each experiment.

At each time point, subsamples were taken for prokaryotic abundance, single-cell respiration and production, DOM and inorganic nutrients and analysed as described elsewhere [20]. In addition, the subsample of the microbial inoculum was collected just prior to the start of both experiments (at 0 h) for bacterial metagenomics and metaproteomics analyses as described below. Subsamples were also taken from each of the flasks at the peak of bacterial abundance (at 32 h, only in Experiment I) and during the decay phase of bacterial growth (at 84 h, only in Experiment II) for bacterial metagenomics and proteomics analyses as described below. Temperature and oxygen concentrations were monitored in each of the flasks throughout the experiment. The temperature was constant and the oxygen concentration never dropped below 80% of saturation as determined using Optical Oxygen Sensor (PreSens Precision Sensing GmbH).

### Metagenomics: sample preparation and data analysis

Nucleic acid extraction, metagenomic DNA library preparation and sequencing were performed as described elsewhere [20] and in the Additional File 1. Raw reads were deposited at NCBI under the accession number PRJNA633735. The metagenome assembly, gene prediction, taxonomic and functional annotation and metagenome assemble genome (MAG) construction pipelines are described elsewhere [20] and in the Additional File 1. Metagenome assemblies are available at the following link (https://figshare.com/s/bc4c0fb59e3d0d3e8aeb).

### Metaproteomics: sample preparation and protein extraction

#### Endo-metaproteomes

For analyses of endo-metaproteomes, biomass was collected onto 0.22-µm pore-size hydrophilic PVDF, Durapore® (Millipore) filters. For the coastal endo-metaproteome, 4 L of the microbial inoculum was collected prior to the start of the experiments. For the endo-metaproteome of the jelly-OM degrading microbial community, 500 mL was collected from each jellyfish treatment (J1, J2 and J3) at the peak of prokaryotic abundance (at 32 h) in Experiment 1 and 1 L at the decay phase (at 84 h) in Experiment 2. For the endo-metaproteome of the control treatments, 2 L was collected from each flask (C1, C2 and C3) at the peak of the bacterial abundance (at 32 h) and 3 L at the decay phase (at 84 h). Protein extraction from collected cells was performed as described in the Additional File 1.

#### Exo-metaproteomes

The 0.2-µm filtrate from each replicate was also collected for exo-metaproteome analysis. The filtrate was concentrated using a VivaFlow 200 with 30,000 Da and 5000 Da Molecular Weight Cut-Off (MWCO) to collect the high molecular weight (30,000 Da – 0.2 µm) and the low molecular weight (5000 Da – 30,000 Da) DOM fraction, respectively. The high and low molecular weight DOM fractions were further concentrated to 250 µL using an Amicon Ultra-15 Centrifugal Filter 30,000 Da MWCO and 3000 Da MWCO Unit (Merck-Millipore). NuPAGE sample reducing agent (Invitrogen) was added to samples to reach 1X final concentration. These samples were stored at − 20 °C until further processing. Details on extraction procedures are provided in the Additional File 1.

### LC–MS/MS analysis: peptide identification and protein enrichment analysis

LC–MS/MS analysis and peptide identification was performed as previously described in detail [50] with slight modifications described elsewhere [20] and briefly summarized in the Additional File 1. The mass spectrometry proteomics data have been deposited to the ProteomeXchange Consortium [51] via the PRIDE [52] partner repository with the dataset identifier PXD036989.

For the metaproteomic analysis of the total community, putative genes were then predicted on contigs longer than 200 bp using Prodigal (2.6.3) [53] and further clustered at 90% similarity (-c 0.9 -G 0 -aS 0.9) using CD-HIT(4.6.8) [54] to construct the prokaryotic gene database for downstream metaproteomic analysis. The MS/MS spectra of proteomic samples were pooled and searched using SEQUEST-HT [55] engines against the protein databases. A target–decoy approach [56] was used in this step and results < 1% FDR at the peptide level were kept. Percolator in Proteome Discoverer 2.1 (Thermo Fisher Scientific) was used for validation. A minimum of two peptides and one unique peptide were required for protein identification. Functional annotation of identified protein sequences was performed by searching against EggNOG [57], KEGG [58] and Pfam [59] databases. Taxonomic affiliation of sequences was determined using the lowest common ancestor (LCA) algorithm adapted from DIAMOND (v2.0.9) blast [60] by searching against the non-redundant (NR) database. SignalP (5.0) [61] was used to detect the presence of signal peptides for extracellular enzymes of bacterial origin.

We conducted protein enrichment analysis for selected MAGs to identify differentially abundant proteins. First, the amino acid sequence database was generated for each MAG separately using prodigal prediction and functional annotations were assigned using KofamKOALA [62]. The raw files from metaproteomic analyses were searched against the corresponding MAG amino acid sequence database with the SEQUEST search engine implemented in Proteome Discoverer. A chromatographic peak area-based label-free quantitative method was employed to obtain relative protein abundances in each metaproteome sample. Relative protein abundances were analysed using the EdgeR package for enrichment analysis [63]. Proteins with log2 fold-change (Log2FC) > 1 and FDR < 0.05 were kept as differentially abundant proteins.

### Statistical analysis

All the statistics and visualization were performed using specific packages in R (https://www.r-project.org/). Vegan, ggplot2 and complex heatmap were used for ordination, diversity calculation and visualization, respectively.

## Results

In the endo-metaproteome fraction (> 0.2 µm) the number of detected proteins were similar among the three biological replicates as well as between different treatments and experiments (~ 10,000 proteins per samples, pre-filtration steps > 1 unique peptides and ≥ 2 peptides were applied; Additional Files 1 and 2, Table S1). Differences in the exo-metaproteomes (< 0.2 µm) were detected, however, with a higher number of proteins detected in the jellyfish treatments (~ 5000 and ~ 1000 proteins in the HMW and LMW fraction (ExoH and ExoL, respectively) as compared to the controls (~ 500 proteins) (Additional File 2, Table S1). This pattern was consistent during late exponential and decay phase. We also screened our exoproteomes for jellyfish proteins (as described previously [20]) revealing that proteins of bacterial origin represented > 90% of all proteins detected in all treatments at all time points.

### Taxonomic origin of the endo- and exo-metaproteomes of the jelly-OM degrading community

**Fig. 1:**
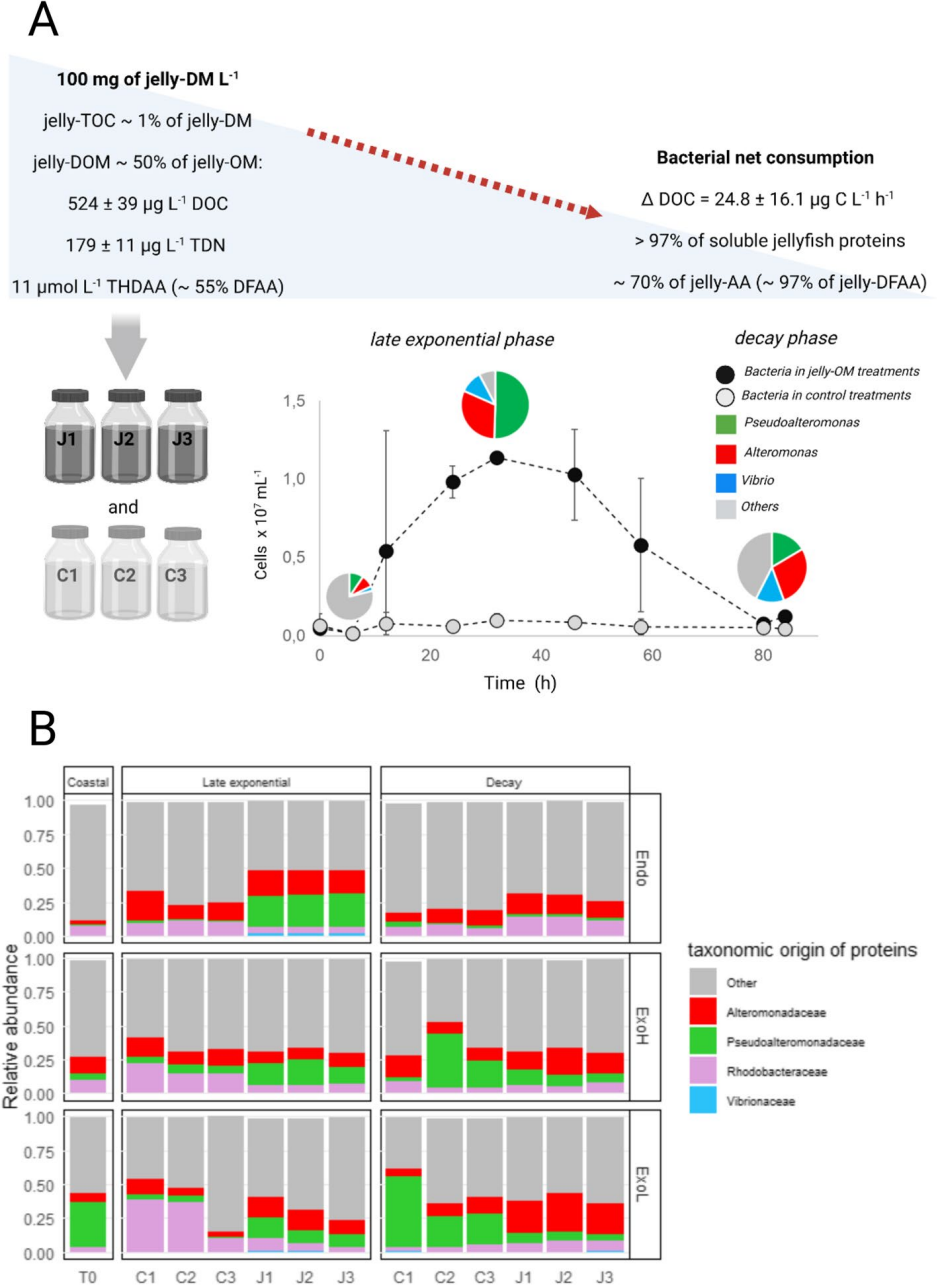
Design of the experiment simulating jellyfish bloom decay, highlighting the key bacterial populations in the jelly-OM degradation process. **A** Experimental design and summary of the bacterial degradation experiment using jelly-OM, highlighting the microscopy-based abundance of major populations involved in the process. **B** Taxonomic origin of proteins detected within the endo- and exo-metaproteomes (ExoH—fraction between 30,000 Da and 0.2 µm, ExoL—fraction between 3000 Da and 30,000 Da) during different phases of bacterial growth (late exponential, decay) in jelly-OM or control treatments and coastal assemblage are depicted. Note: in *y*-axes 1 equals 100%. Dominant taxa are highlighted: *Alteromonadaceae*—red, *Pseudoalteromonadaceae*—green, *Vibrionaceae*—blue, *Rhodobacteraceae*—pink, DM-dry matter; DOM—dissolved organic matter; TOC—total dissolved carbon, DOC—dissolved organic carbon, TDN—total dissolved nitrogen; AA—amino acids; THDAA—total hydrolysable dissolved amino acids; DFAA—dissolved free amino acids; J—jelly-OM treatments, C—controls

The endo-metaproteome of the microbial community used to set up the enrichment experiment was dominated by Alphaproteobacteria (~ 41%), followed by Gammaproteobacteria (~ 21%) (Additional File 3: Fig. S1). In contrast, the exo-metaproteome was dominated by Gammaproteobacteria, representing ~ 46% of all proteins in the HMW fraction (ExoH, 30,000 Da – 0.2 µm) and ~ 78% in the LMW fraction (ExoL, 5000 Da – 30,000 Da), while Alphaproteobacteria accounted for ~ 40 and ~ 15% in the ExoH and ExoL fraction, respectively (Additional File 3: Fig. S1). Exposing this coastal assemblage to jelly-OM, the structure of the microbial community [20] and hence the taxonomic origin of the detected proteins changed rapidly (Fig. 1, Additional File 3: Fig. S1).

Already after 32 h, when the jelly-OM degrading community reached late exponential growth phase, the relative abundance of gammaproteobacterial proteins significantly increased as compared to the control treatments (to 74.9 ± 0.4% of all detected proteins, Welch’s *t*-test, *p* < 0.05) (Additional File 2: Table S2, Additional File 3: Fig. S1). At the same time, the relative contribution of Alphaproteobacteria decreased in the jelly-OM and control incubations as compared to coastal seawater (11.7 ± 0.3% and 27.1 ± 2.3%, respectively; Additional File 2: Table S2, Additional File 3: Fig. S1). The endo-metaproteomes of the jelly-OM degrading community were significantly enriched in gammaproteobacterial proteins from *Pseudoalteromonadaceae* (23.9 ± 0.9%, *p* < 0.001) and Vibrionales (4.0 ± 0.3%, *p* < 0.01, from which *Vibrionaceae* contributed 1.6 ± 0.1%, *p* < 0.001) as compared to the control treatments (Fig. 1B, Additional File 2: Table S2, Additional File 3: Fig. S1). However, there was no significant difference in the relative contribution of *Alteromonadaceae* proteins between the endo-metaproteomes of the jelly-OM and control treatments (Additional File 2: Table S2, Additional File 3: Fig. S1). The exo-metaproteomes of the jelly-OM treatment were significantly enriched in *Pseudoalteromonadaceae* proteins compared to the control incubations representing 15.3 ± 2.0% (*p* < 0.05) and 11.2 ± 2.2% (*p* < 0.05) of all detected proteins in the ExoH and the ExoL fraction, respectively (Fig. 1, Additional File 2: Table S2).

Once the bacterial community in the jelly-OM treatments reached its decay phase (after 84 h), the relative abundance of gammaproteobacterial proteins decreased to 45.3 ± 5.5% in the jelly-OM endo-metaproteomes and there was no significant difference in the taxonomic origin of proteins detected between the jelly-OM treatment and the unamended controls at the class level (Additional File 2: Table S2, Additional File 3: Fig. S1). However, at the order level, proteins of Alteromonadales were still abundant in the jelly-OM endo-metaproteomes (20.4 ± 2.5%, *p* < 0.05) (Additional File 2: Table S2, Additional File 3: Fig. S1). Overall, the taxonomic diversity of endo-metaproteomes increased within the jelly-OM treatments during the decay phase as compared to the late exponential phase with high relative abundances of alphaproteobacterial proteins (32.2 ± 4.5%) (Additional File 2: Table S2, S3). Proteins of Rhodobacterales were significantly enriched (18.4 ± 0.25%, *p* < 0.05) in the endo-metaproteomes of the jelly-OM treatment and were mostly assigned to *Rhodobacteraceae* (12.9 ± 1.5%, *p* < 0.01) (Fig. 1, Additional File 2: Table S2, Additional File 3: Fig. S1). In the ExoH fraction, gammaproteobacterial proteins affiliated with Oceanospirillales within the order Alteromonadales, family *Colwelliaceae* (4.1 ± 1.3%, *p* < 0.05 and 1.5 ± 0.6%, *p* < 0.05, respectively) were significantly enriched in jelly-OM *vs* control treatments (Additional File 2: Table S2, Additional File 3: Fig. S1). In the ExoL fraction, proteins affiliated with *Alteromonadaceae* (25.2 ± 3.3%, *p* < 0.01) and *Rhodobacteraceae* (7.5 ± 1.0%, *p* < 0.05) were significantly enriched in jelly-OM exo-metaproteomes (Fig. 1, Additional File 2: Table S2, Additional File 3: Fig. S1).

### Functional profiles of endo- and exo-metaproteomes of the jelly-OM degrading community

**Fig. 2:**
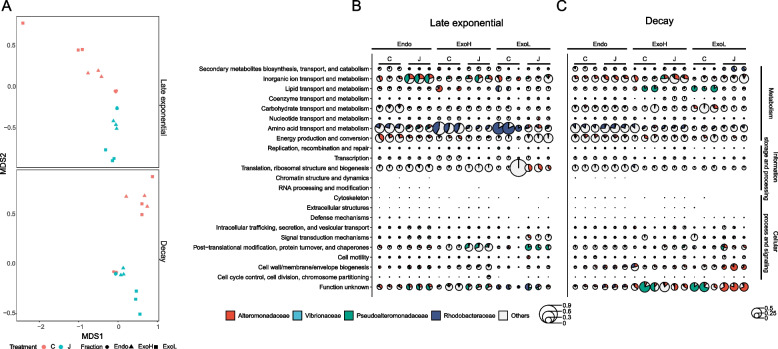
Clustering of the jelly-OM degrading community metaproteomes at orthologous groups level.** A** NMDS of proteins clustered at orthologous groups (OG) level detected in the endo- and exo-metaproteomes of jelly-OM degrading microbial communities (J) and in the control (C) treatments in the late exponential and decay growth phase. **B,C** Clusters of orthologous groups (COG categories) enriched in jelly-OM over control treatments during late exponential (**B**) and decay (**C**) phase of bacterial growth. The taxonomic origin of proteins clustered per COG category is shown. Only COG categories representing at least 1% relative abundance of all proteins detected in at least one sample are depicted. Size of a circle corresponds to relative abundance of specific COG category

Non-metric multidimensional scaling (NMDS) analysis based on orthologous groups (OGs) revealed that the addition of jelly-OM significantly changed the functional profiles of the microbial community during late exponential growth, particularly in the ExoL fraction (Fig. 2A, PERMANOVA, *p* < 0.01). When the jelly-OM amended bacterial community entered the decay phase, only profiles of exo-metaproteomes (ExoL and ExoH fraction) were significantly different from those of the controls (Fig. 2A).

By grouping proteins according to clusters of orthologous groups (COG) and linking them to the taxonomic composition (Fig. 2B), we found significant enrichment in certain COG categories in the endo-metaproteomes of the jelly-OM degrading community during late exponential growth (Additional File 2: Table S4, S5). Particularly, proteins involved in inorganic ion transport and metabolism (22.9 ± 0.9% of all proteins, *p* < 0.0001), mostly affiliated with *Pseudoalteromonadaceae*, became the predominate function in the endo-metaproteome of jelly-OM degrading community (Fig. 2B, Additional File 2: Table S4, S5). Post-translational modification, protein turnover, and chaperones (15.7 ± 1.1%, *p* < 0.05) assigned to *Pseudoalteromonadaceae* were the main enhanced functions in the ExoH and Exo-L fractions of the jelly-OM treatments during the late exponential phase (Fig. 2B, Additional File 2: Table S4, S5). In contrast, the relative abundance of proteins involved in amino acid metabolism decreased after jelly-OM amendment with the decrease of *Rhodobacteraceae* in the treatment (Fig. 2B, Additional File 2: Table S4, S5). The relative abundance of most endo-metaproteome functions enhanced by jelly-OM addition in the late exponential phase decreased once bacteria entered the decay phase (Fig. 2B).

Yet, some functions remained enriched in the exoproteomes from jelly-OM treatments. These included inorganic ion transport and metabolism (18.3 ± 0.4%, *p* < 0.05) proteins of *Alteromonadaceae*, amino acid transport and metabolism (11.3 ± 1.5%, *p* < 0.05) proteins of *Rhodobacteraceae*, and proteins associated with post-translational modification, protein turnover and chaperones (5.7 ± 0.1%, *p* < 0.05) of *Pseudoalteromonadaceae* (Fig. 2B, Additional File 2: Table S4, S5).

#### Proteases and carbohydrate-active enzymes of the jelly-OM degrading community

**Fig. 3:**
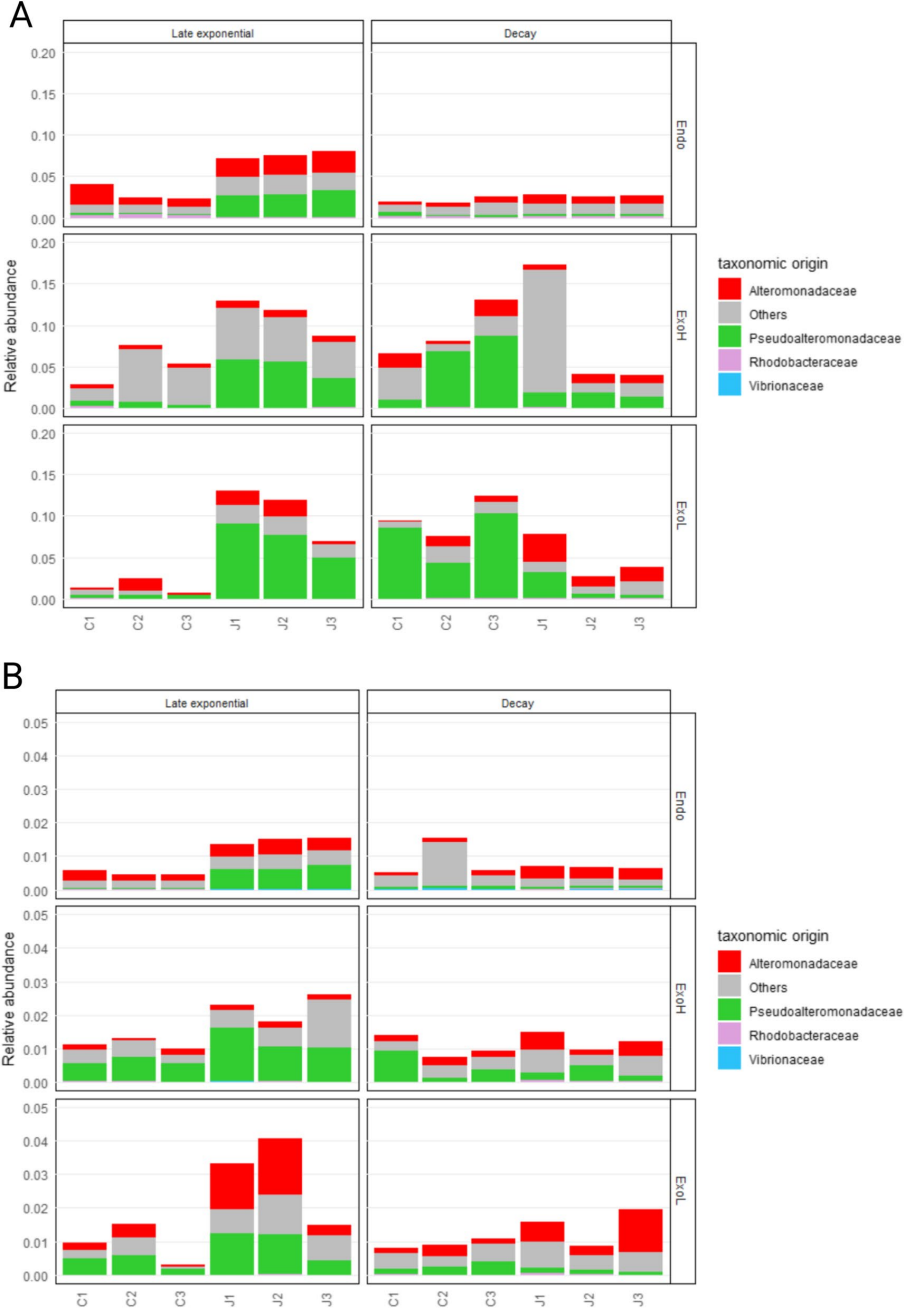
Relative abundance and taxonomy of proteases and CAZymes in the metaproteomes of jelly-OM degrading community. Protease (**A**) and CAZymes (**B**) are expressed as percentage of total proteins detected in the endo- and exo-metaproteomes during different phases of bacterial growth in the jelly-OM (J) and control (C) treatments. For detailed information, see Additional File 2: Table S6 and S8 and Additional File 3: Fig. S2. Note: in *y* axes 1 equals 100%. Note the differences on *y*-axes scale

To explore how the microbial community remineralizes jelly-OM, we focused on the functional composition and taxonomic origin of proteases and carbohydrate-active enzymes (CAZymes). Overall, proteases were significantly enriched in all three proteomic fractions after jelly-OM amendment as compared to the control treatment in the late exponential phase (Fig. 3A, Additional File 2: Table S6, Welch’s *t*-test). The highest relative abundance of proteases was found in the exoproteome fraction (ExoH: 11.2 ± 2.2%; ExoL:10.6 ± 3.2% of all proteins, *p* < 0.05), followed by endo-metaproteomes (7.5 ± 0.4% of all proteins, *p* < 0.01) (Additional File 2: Table S6). Signal peptide analysis indicated that > 70% of all proteases in the ExoH and ExoL fraction were secretory in our dataset (Additional File 2: Table S7). Proteins containing signal peptides and were found in the exoproteome fraction (< 0.2 µm) were considered as secretory enzymes in our study; however, note that proteins release from dead cells cannot be excluded. The most abundant proteases were serine proteases with the S8 family (mainly S8A subfamily, Subtilisin) accounting for ~ 30, ~ 66 and ~ 54% of all proteases in the endo-, exoH- and exoL-proteomes of the jelly-OM treatments, respectively (Additional File 2: Table S6, Additional File 3: Fig. S2). *Alteromonadaceae* (~ 40%), followed by *Pseudoalteromonadaceae* (~ 17%) were the major sources of S8 proteases in the endo-proteomes of the jelly-OM treatments and *Pseudoalteromonadaceae* dominated the S8 protease in the ExoH and the ExoL fraction (~ 48 and ~ 94% of S8 proteases, respectively) (Fig. 3A, Additional File 2: Table S6). Metalloproteases were the second most abundant protease family produced by jelly-OM degraders, among which the M9 family was the major metalloprotease produced almost exclusively by *Pseudoalteromonadace*, representing ~ 1% and ~ 2% of all proteases in the endo- and exoH fraction during late exponential phase, respectively (Additional File 2: Table S6; Additional File 3: Fig. S2). Protease inhibitors (~ 100% I39 family of *Pseudoalteromonadaceae* and *Alteromonadaceae* origin) were significantly enriched in the jelly-OM endo-metaproteomes during late exponential growth, representing 14.9 ± 0.06% (*p* < 0.001) of all proteins, but no significant differences between treatments were detected in the exo-metaproteomes (Additional File 2: Table S6). Once the jelly-OM degrading community entered the decay phase, endo- and exo-metaproteomes in the jelly-OM treatment were similar to the unamended control with the exception of protease inhibitors (I39 family, predominantly of *Pseudoalteromonadaceae*), which were enriched during the decay phase in the ExoH fraction of the jelly-OM degrading community (6.3 ± 0.7%, *p* < 0.05, of all proteins) (Additional File 2: Table S6).

CAZymes were also enriched in the metaproteomes of the jelly-OM treatment, representing 1.5 ± 0.1% of the total proteins (*p* < 0.001) in endo- and 2.2 ± 0.4% of total proteins in the ExoH fraction of the exo-metaproteomes (*p* < 0.05) during late exponential growth (Fig. 3B, Additional File 2: Table S8). However, their relative abundance was considerably lower (~ 5 times) than proteases (Fig. 3B). Similar to proteases, *Pseudoalteromonadaceae* and *Alteromonadaceae* were the major sources for CAZymes in all three fractions, with the exception that *Alteromonadaceae* contributed more to the CAZymes in the ExoL fraction than *Pseudoalteromonadaceae* (Fig. 3B, Additional File 2: Table S8). The endo-metaproteomes of jelly-OM treatments were significantly enriched in carbohydrate esterases (CEs), glycoside hydrolases (GHs), glycosyl transferases (GTs), polysaccharide lyases (PLs), and enzymes annotated as auxiliary activity class (AAs, i.e. redox enzymes that act in conjunction with CAZymes), while the ExoH fraction of exo-metaproteomes was exclusively enriched in GHs during late exponential growth (Additional File 2: Table S8; Additional File 3: Fig. S2). The relative abundance of CAZymes decreased as the jelly-OM degrading bacterial community entered the decay phase (Fig. 3B). The functional and taxonomic profiles of CAZymes in the decay phase were similar to the control treatment, except for AAs from *Rhodobacteraceae* (*p* < 0.01, but below 0.01% of relative abundance) and *Alteromonadaceae* (*p* < 0.05) which still exhibited high relative abundances in the jelly-OM endo- and exoL-proteomes, respectively (Fig. 3B, Additional File 2: Table S8; Additional File 3: Fig. S2).

### Protein profiles of key taxa dominating the jelly-OM degrading consortium

Our metagenome analysis coupled with previous microscopy-based analysis pointed to the key role of *Pseudoalteromonadaceae* and *Vibrionaceae* populations during the initial jelly-OM degradation and the potential importance of *Alteromonadaceae* and *Rhodobacteraceae* populations during the decay phase of jellyfish blooms (Figs. 1, 2, and 3; Table 1; [20]). We binned five metagenome-assembled genomes (MAG) of key taxa within jelly-OM degrading community: one *Alteromonas* (MAG29), two *Pseudoalteromonas* (MAG22 + MAG24), one Vibrio (MAG3) and one *Thalassobius* (MAG51), representing 43, 39, 7, and 3% of all metagenomics reads mapped to total MAGs in the jelly-OM treatment during late exponential growth, respectively (Table 1).

**Table 1:** Overview of representative MAGs of key populations within the jelly-OM degrading consortium. The taxonomic affiliation of MAGs was inferred from GTDB-TK classification results. Details on annotated genes and proteins for each MAG can be found in Additional File 2: Table S15

	**Taxonomy**								**% of all mapped reads**			
MAG ID	Family	Genus	Closest placement_ani	Est. comp	Est. cont	GC content (%)	N50	Assem. size (Mbp)	Coastal	Control	Jelly-OM	No. of annotated proteins in our dataset/ No. of Kegg annotated genes in our dataset/ No. of CDSs of closest relative/placement
3	Vibrionaceae	**Vibrio**	GCF_000222625.1 (Vibrio splendidus)	98.49	3.66	44.2	84,942	5.13	**0.01**	**0.33**	**6.5**	**356**/2742/4462
22	Alteromonadaceae	**Pseudoalteromonas**	GCF_001444405.1 (Pseudoalteromonas phenolica)	87.48	4.11	41	22,419	4.65	**0.12**	**0.16**	**23.1**	**656**/2004/4154
24	Alteromonadaceae	**Pseudoalteromonas**	GCF_001444405.1 (Pseudoalteromonas phenolica)	60.41	4.297	40.4	15,108	3.98	**0.06**	**0.15**	**16.1**	**485**/1654/4154
29	Alteromonadaceae	**Alteromonas**	GCF_000172635.2 (Alteromonas macleodii)	99.52	4.035	42.9	221,177	4.63	**0.00**	**14.2**	**42.8**	**900**/2209/3883
51	Rhodobacteraceae	**Thalassobius**	GCF_900156595.1 (Thalassobius mediterraneus)	92.29	1.384	58.9	191,610	3.14	**0.01**	**0.48**	**3.3**	**263**/1799/3218

**Fig. 4:**
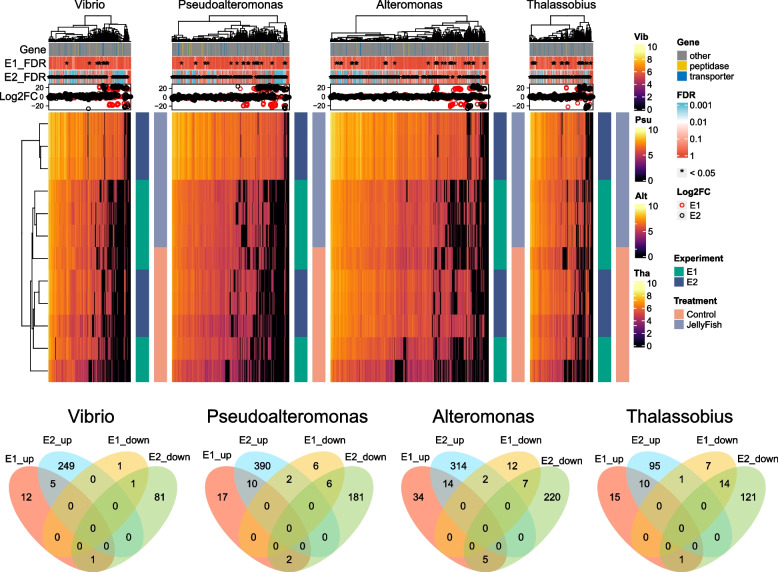
Profiles of differentially abundant proteins of key MAGs constituting jelly-OM degrading consortium. *Top panels*: Gene: the total gene profile of each MAG: genes encoding peptidases are in yellow, genes encoding transporters in blue, the rest is shown in grey. The false discovery rate (FDR) of log2 transformed fold-change (Log2FC) of all proteins per MAG after jelly-OM amendments during the late exponential (E2_FDR) and decay (E1_FDR) phase, FDR < 0.05 is highlighted using (*). Log2FC of individual proteins during late exponential (black dots) and decay phase (red dots) is presented. In the heatmap, jellyfish treatments are highlighted as bars in blue, in orange the control treatments, late exponential phase in dark blue (E2), and decay phase in green (E1). *Bottom panels*: Numbers of differentially abundant proteins for which relative abundance increased (up) or decreased (down) exclusively during late exponential (E2) and/or decay phase (E1) and/or throughout the jelly-OM degradation process are also presented as Venn diagrams. Only one of the *Pseudoalteromonas* MAGs is depicted, for other with almost identical pattern see Additional File 3 Fig. S3. For details see Additional File 2: Table S9

**Fig. 5:**
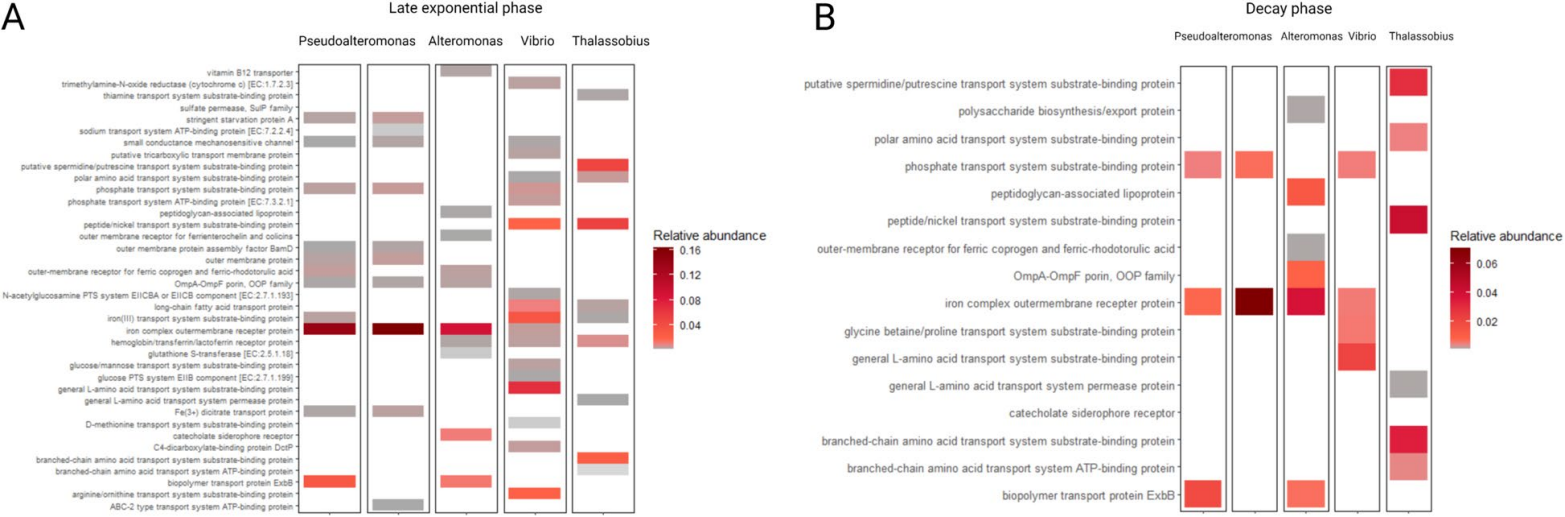
Transporters associated with key taxa during different stage of jelly-OM degradation. Transporters (> 0.1% of relative abundance) of *Pseudoalteromonas* (MAG22 and MAG24), *Alteromonas* (MAG29), *Vibrio* (MAG3), and *Thalassobius* (MAG51) during late exponential (**A**) and decay (**B**) phase and their relative abundance shown as mean values of biological replicates. For details on all transporters associated with each MAG, please see Additional File 2: Table S10. Note: in *y* axes 1 equals 100%

**Fig. 6:**
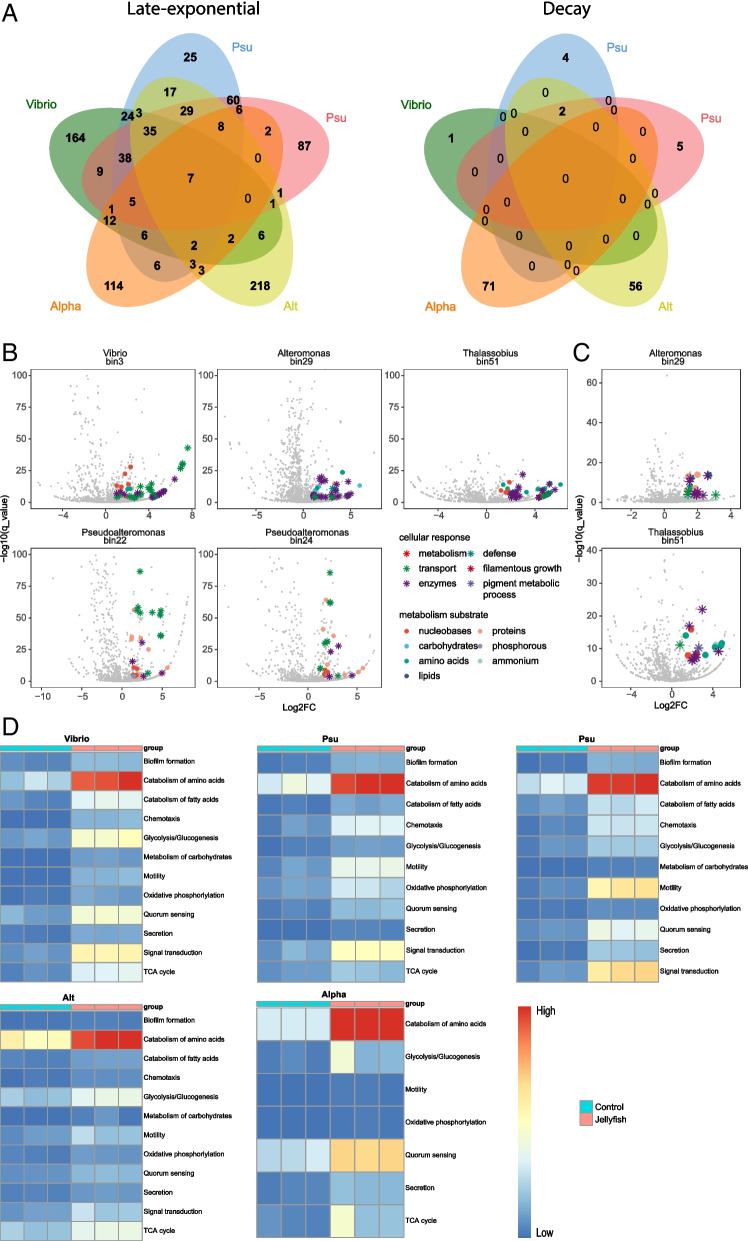
Shared and unique metabolic processes mediated by key taxa during the jelly-OM degradation process. **A** Number of upregulated GO terms in jelly-OM vs. control treatment associated with specific MAGs and shared among different key MAGs of jelly-OM degrading consortium during late exponential and decay phase of bacterial community growth. **B, C** Log2FC and q-value of up- and down-regulated GO terms during late exponential (**B**) and decay (**C**) phase of bacterial community growth: highlighted are metabolic processes associated with specific substrate and some specific features associated with cellular response to jelly-OM amendment. **D** Heatmap of selected individual proteins (KEGG annotated) grouped according to their role in interactions within the microbial community and environmental information processing and specific catabolic/metabolic pathways. For details, please see Additional File 2: Table S11, S12

**Fig. 7:**
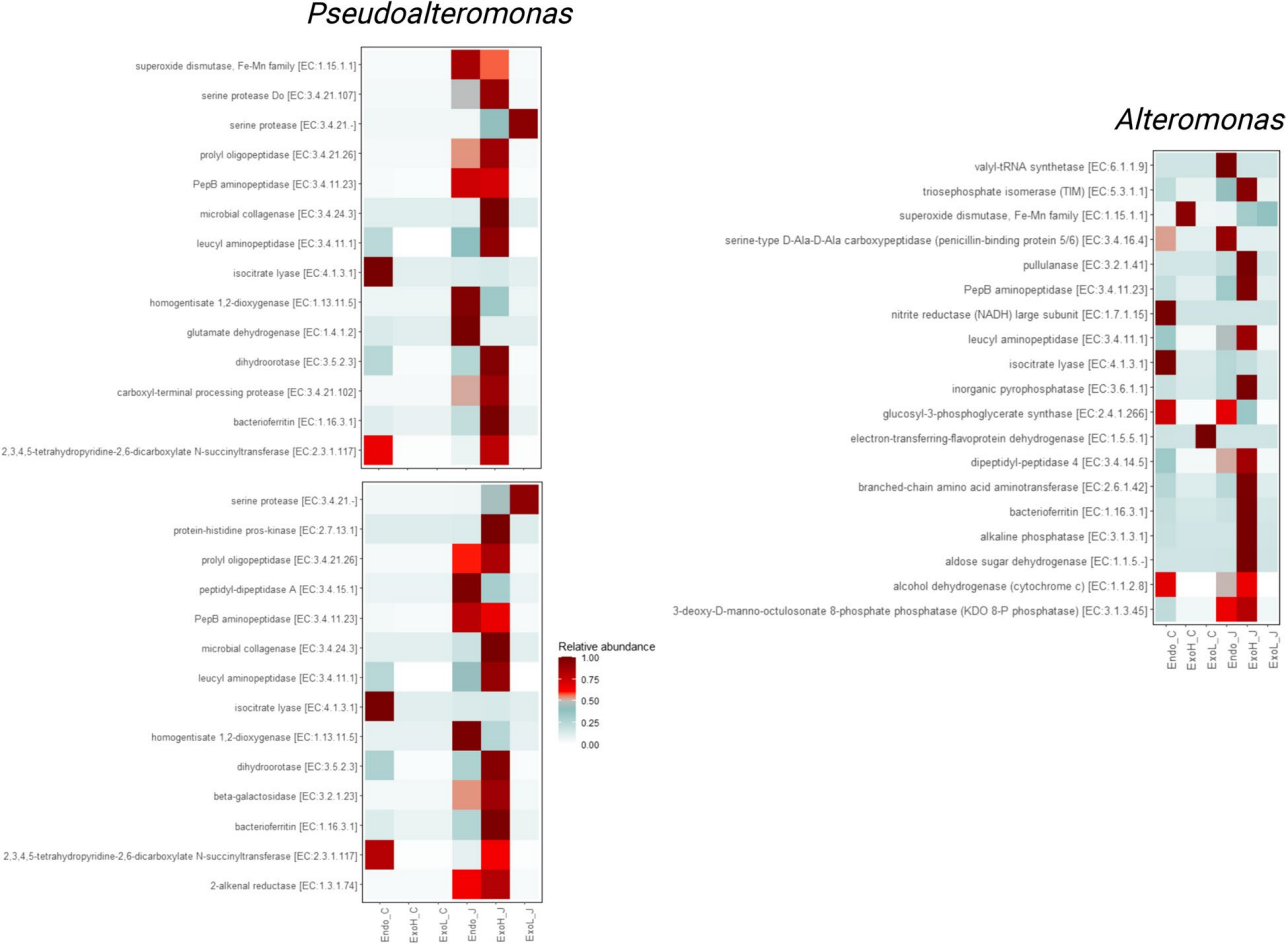
Exoproteome of key jelly-OM degraders. Relative abundance of exoproteins (in the ExoH and ExoL fraction) and their intracellular counterparts (Endo) during late exponential phase of bacterial community growth in the jelly-OM (J) and control (C) treatments. In the exoproteomes, only extracellular enzymes associated with *Pseudoalteromonas* and *Alteromonas* were detected. For details, see Additional File 2: Table S14. Note: Note: in y axes 1 equals 100%

Here, we decipher the metabolic response of these five taxa, represented by corresponding MAGs, to jellyfish-OM addition. First, we analysed changes in the endo-metaproteome profiles of different representatives of key populations (Fig. 4), focusing on the transport proteins synthesized during different stages of jelly-OM degradation by different bacteria (Fig. 5) and on key metabolic pathways of each representative of the jelly-OM degrading consortium (Fig. 6). We assumed that higher relative abundances of proteins related to specific transport and metabolism were triggered by the availability of a substrate, although it might as well be the opposite, e.g. enrichment due to the lack of specific resources ([45] and the references therein). We also analysed proteins affiliated with specific MAGs in our exo-metaproteome dataset during different stages of jelly-OM degradation (Fig. 7).

#### Endo-metaproteome profiles of key jelly-OM degraders

The five key taxa exhibited a substantial metabolic response after jelly-OM amendment during the late exponential phase of bacterial growth (Fig. 4, Additional File 3: Fig. S3, S4). More than 90% of the detected proteins were differentially abundant in *Pseudoalteromonas*, *Vibrio* and *Thalassobius* and ~ 68% in *Alteromonas* (Table 1, Fig. 4; Additional File: Fig. S3). A higher percentage of proteins increased in relative abundance in the late exponential (~ 58%) than in the decay phase (~ 4%) (Fig. 4, Additional File 3: Fig. S3, S4, Additional File 2: Table S9). Yet, differences in endo-metaproteome profiles between jelly-OM and control treatments were more pronounced for *Pseudoalteromonas* and *Vibrio* as compared to *Alteromonas* and *Thalassobius* during late exponential growth (Fig. 4). In *Pseudoalteromonas* and *Vibrio*, we detected a higher percentage of proteins with increased relative abundance (63, 67 and 71%, respectively) and a lower percentage of proteins with decreased relative abundance (30, 24 and 23%, respectively) during late exponential growth phase as compared to *Alteromonas* and *Thalassobius* (52 and 36% increased relative abundance, 36 and 45% decreased relative abundance, respectively) (Fig. 4, Additional File 3: Fig. S3, S4, Additional File 2: Table S9).

When the bacterial community entered its decay phase, differences in endo-metaproteome profiles between the two treatments decreased for *Pseudoalteromonas* and *Vibrio*. In contrast, the endo-metaproteome profile of *Alteromonas* and *Thalassobius* remained distinct in the jelly-OM compared to the control treatment (Fig. 4, Additional File 3: Fig. S3, S4, Additional File 2: Table S9).

##### Transporters and associated proteins

Proteins associated with transport across the cell membrane were highly abundant in all key taxa during late exponential phase (Fig. 5, Additional File 2: Table S10). During this stage of jelly-OM degradation, proteins associated with scavenging, chelating, and transport of iron across the cell membrane were shared among all MAGs (Fig. 5, for details see Table S10, i.e. group of TonB transporters encoded with genes *fecA*, *fhuE, fpvA, fptA, TC.FEV.OM*, [64]). Instead, siderophores-specific transporters were associated only with gammaproteobacterial MAGs (Additional File 2: Table S10). Phosphate transporters were only associated with *Pseudoalteromonas* and *Vibrio* (Fig. 5A, Additional File 2: Table S10). *Vibrio* and *Thalassobius* also produced transporter proteins for the transport of peptide/nickel, amino acids, long-chain fatty acid, and putrescine. Some transporters were also unique to each taxon. For example, *Pseudoalteromonas* expressed genes encoding for small conductance mechanosensitive ion channel proteins, ABC-type Na^+^ transporters as well as a transporter system for taurine (NiT/TauT transporter with < 0.1% relative abundance, Additional File 2: Table S10) (Fig. 5A). *Vibrio* expressed transporters for different amino acids (e.g. arginine/ornithine, D-methionine, glycine betaine/proline transport system, with < 0.1% relative abundance, Table S10) and carbohydrates (e.g. glucose PTS system, glucose/mannose transport system, N-acetylglucosamine PTS system) (Fig. 5A). *Alteromonas* synthesized transporters for vitamin B_12_ and organophosphorus compounds (phosphonate) (Fig. 5A, Additional File 2: Table S10). In contrast to the gammaproteobacterial taxa, the alphaproteobacterial taxon *Thalassobius* expressed transporters for thiamine/vitamin B_1_ and branched-chain amino acids (Fig. 5A, Additional File 2: Table S10).

When the bacterial community entered its decay phase, the protein abundance pattern of each taxa changed and overall, the relative abundance of transporter proteins decreased (Fig. 5B). However, *Thalassobius* produced a more diverse set of transport systems than gammaproteobacterial taxa at this stage, particularly proteins associated with iron transport and phosphate uptake as well as transporters for peptides, amino acids, putrescine/spermidine, and vitamin B_1_ (Fig. 5B, Additional File 2: Table S10).

##### Intracellular metabolic processes mediated by key jelly-OM degraders

Only seven enriched GO terms were shared by the five key MAGs of the jelly-OM degrading consortium during late exponential growth, associated with alpha-amino acid catabolic processes, iron scavenging, and different types of homeostatic processes (Fig. 6A, B, Additional File 2: Table S11). Nevertheless, significant differences in the associated GO enrichment (log2 fold-change, hereinafter log2FC) were found among key jelly-OM degraders (Fig. 6 for catabolism of amino acids, Additional File 2: Table S11). For example, GO terms related to biosynthesis and metabolism of siderophores were mainly detected in *Pseudoalteromonas* (log2FC ~ 4.5, q < 2e − 05) and to less extend in *Alteromonas* (log2FC ~ 1, q < 7e − 06), while all key gammaproteobacterial taxa (including *Vibrio*) produced proteins for the transport and uptake of siderophores during late exponential growth (Additional File 2: Table S11).

##### Interactions within the microbial community and environmental information processing

**Fig. 8:**
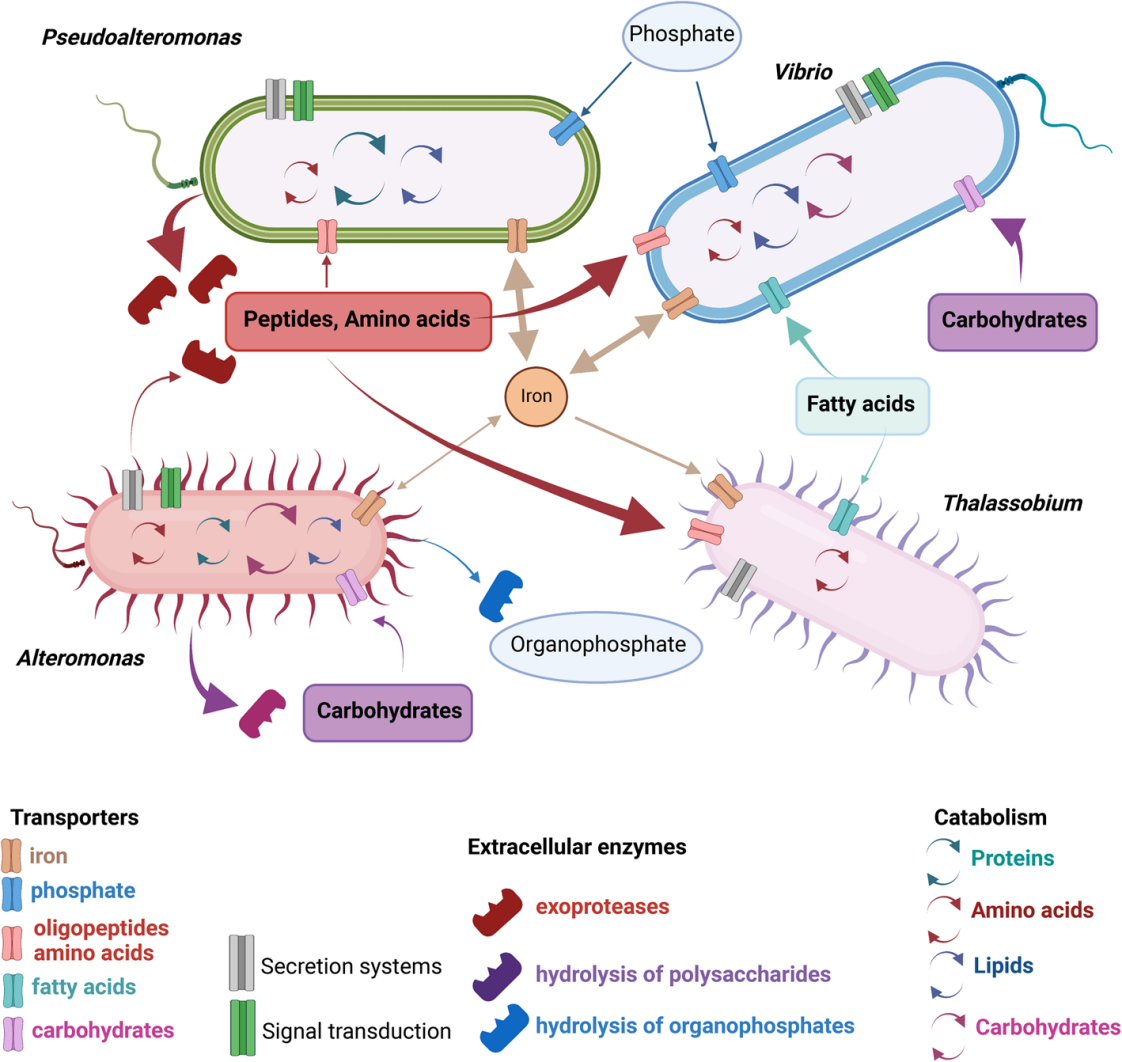
Niche partitioning and metabolic interactions within the metabolic network operated by key jelly-OM degraders

During late exponential growth phase, all representatives of key jelly-OM degraders expressed genes for flagella or pilus (e.g. K02650, K03406, K0407, K03408, K02280) and synthesized proteins involved in quorum sensing (e.g. K01897, K10912, K14645, K03071, K03076, K03666, K02055, K03070) and secretion systems (K03070, K03071, K03076, K02453) (Figs. 6C and 8, Additional File 2: Table S12). However, the repertoire and abundance level of individual proteins associated with motility, quorum sensing, and secretion systems differed among the representatives of the key taxa in the jelly-OM treatments (Figs. 6C and 8, Additional File 2: Table S12). For example, in *Pseudoalteromonas*, motility-related proteins showed a higher relative abundance in jelly-OM than in the control treatments compared to other taxa (Fig. 6C, Additional File 2: Table S11, S12). The synthesis of quorum sensing proteins was less pronounced in *Alteromonas* as compared to other taxa during late exponential growth in the jelly-OM treatments (Fig. 6C, Additional File 2: Table S11, S12). In the jelly-OM treatments, only gammaproteobacterial taxa, particularly *Pseudoalteromonas* and *Vibrio*, produced proteins for chemotaxis (e.g. K03406, K03407, and K03408), biofilm formation (e.g. K02453, K10912, K20978, K03666), and signal transduction (e.g. K00405, K00411, K00412, K02650, K03406, K03407, K03408, K10912, K20978, K07795, K07811, K11688) (Fig. 6C, Additional File 2: Table S12). Proteins associated with defense mechanisms were unique to *Alteromonas* (e.g. GO:0042742, GO:0050829, log2FC ~ 4, q < 10e − 10) and *Thalassobius* (e.g. GO:0071236, GO:0097237, log2FC ~ 2, q < 1e − 06). The latter also expressed genes associated with invasive filamentous growth (GO:0030447, GO:0044182) during late exponential growth (Fig. 7B, C, Table S11).

##### Metabolism/catabolism of proteins and amino acids

During late exponential growth, we detected higher relative abundances of endopeptidases (i.e. breaking down peptide-bonds of non-terminal amino acids required for the first step of the intracellular hydrolysis of proteins) in all key gammaproteobacterial taxa (Additional File 2: Table S13, Additional File 3: Fig. S5). Analysis at the individual protein level revealed shared and unique sets of endopeptidases synthesized by each representative of key jelly-OM degraders (Additional File 2: Table S13 Additional File 3: Fig. S5). Notably, both *Pseudoalteromonas* genera synthesized a substantially larger array of different endo-proteases than other taxa and some even exclusively, such as microbial collagenases (K01387, EC: 3.4.24.3), serine proteases (e.g. K14645, EC: 3.4.21.-), and prolyl oligopeptidases (K01322, EC: 3.4.21.26) (Additional File 3: Fig. S5, Additional File 2: Table S13). Based on GO enrichment analysis, catabolism of collagen was unique to *Pseudoalteromonas* (GO:0030574, GO:0032963, log2FC ~ 5, q-value < 1e − 08, Additional File 2: Table S11). Among all key jelly-OM degraders, protein and amino acid metabolism/catabolism was also most profoundly enriched in *Pseudoalteromonas* (GO:0043171, GO: 0051603, log2FC ~ 4, q-value < 1e − 08 and GO:1,901,606, log2FC ~ 6.3, q-value < 1e − 35, respectively) (Fig. 6B, C, Additional File 2: Table S11). Additionally, using the KEGG Mapper tool [65, 66], we detected different enzymes, pathways involved in deamination of amino acids, TCA cycle, oxidative phosphorylation, and partly reconstructed pathways involved in the degradation of different amino acids in all jelly-OM degraders (Additional File 2: Table S12).

When the bacterial community entered decay phase, catabolic/metabolic processing of proteins and amino acids was enriched only in *Alteromonas* and *Thalassobius* in jelly-OM treatments (Fig. 6A, B, Additional File 2: Table S11, 12).

##### Catabolism of carbohydrates, lipids, and other types of metabolism

In contrast to protein metabolism, carbohydrate metabolism was associated mostly with *Alteromonas* (e.g. catabolism of polysaccharides (GO:0000272, log2FC ~ 3, q-value 1e − 09), and glycolysis/gluconeogenesis (K00382)) and with *Vibrio* (e.g. fructose and mannose (K01524), and nucleotide sugar metabolism (GO:0009225), glycolysis/gluconeogenesis (K00382, K00627, K01007, K01624)) during late exponential growth (Fig. 6B, C, Additional File 2: Table S11, S12). Fatty acid degradation was most significantly enriched in *Pseudoalteromonas* and *Vibrio*, but was present also in *Alteromonas* (Fig. 6B, C, Additional File 2: Table S11, S12)*.* Metabolism of nucleobases was only maintained by *Alteromonas* and *Thalassobius* during the decay phase (e.g. GO:0034655) although all jelly-OM degraders showed an upregulation during the late exponential growth phase (Fig. 6A, B, Additional File 2: Table S11, S12).

#### Exo-metaproteome profiles of key jelly-OM degraders

In the exo-metaproteomes of the jelly-OM treatments, we exclusively detected enzymes assigned to *Pseudoalteromonas* and *Alteromonas* (Fig. 7, Additional File 2: Table S14). The majority of enzymes excreted by *Pseudoalteromonas* were proteases including microbial collagenase, serine proteases, and prolyl oligopeptidase (Fig. 7, Additional File 2: Table S14). The exo-metaproteome composition of *Alteromonas* was quite different with no collagenases or serine proteases detected (Fig. 7, Additional File 2: Table S14). *Alteromonas*, however, exclusively secreted enzymes potentially involved in the degradation of branched-chain amino acids (i.e. branched-chain amino acid aminotransferase potentially involved in valine, leucine and isoleucine degradation), hydrolysis of polysaccharides (i.e. pullulanase), and simple sugars (aldose sugar dehydrogenase) and enzymes associated with degradation of organophosphorus compounds (e.g. inorganic pyrophosphate involved in phosphonate degradation) and alkaline phosphates (Fig. 7; Additional File 2: Table S14).

## Discussion

### Decay of jellyfish blooms causes re-shuffling of ambient microbial exoproteomes

Previous studies demonstrated that 20–30% of seawater DOM is composed of high molecular weight (HMW, ≥ 1 kDa) compounds rich in carbohydrates and to lesser extent of lipids and proteins [35, 67–69]. Recently, it has been reported that ~ 80% of labile DOC in the ocean is of phytoplankton origin (from alive, dead, and dying/senescent organisms) and only ~ 20% of heterotrophic organisms (i.e. released by excretion and death process of other microbes, protists, or zooplankton) [36, 47, 48]. So far, one source of DOC has been largely ignored and only recently recognized as knowledge gap – gelatinous zooplankton [24, 29, 47]. Hitherto, it was shown that decaying gelatinous zooplankton blooms cause episodic/seasonal distortion to the ambient seawater DOM and nutrient pool by delivering substantial pulses of HMW proteinaceous compounds, free amino acids, and inorganic nutrients, thus effecting functioning and biogeochemistry of ecosystems [9, 20, 29, 70].

Our multi-omics approach showed that jellyfish detritus triggers a rapid change in the composition and metabolism of the ambient microbial community, revealing a key role of Gammaproteobacteria, predominantly *Pseudoalteromonadaceae*, in the degradation of jelly-OM (Figs. 1, 2, and 3, Additional File 2: Table S2, S3). This is different to the microbial response to phytoplankton-derived OM, where Alphaproteobacteria and/or Bacteroidetes (*Flavobacteria*) outcompete Gammaproteobacteria by scavenging HMW compounds, monomers, and metabolites (from amino acids to organic sulphur compounds, poly- and mono-saccharides) [45, 49, 71, 72].

Our metaproteomic analysis showed that exposure to jelly-OM causes pronounced functional changes in the microbial proteomes, which are most pronounced in the exoproteome fraction, where cell-free enzymes carry out the hydrolytic activity of complex compounds (Fig. 2A). Jelly-OM is mostly composed of complex proteins (e.g. structural and elasticity proteins, like collagen ~ 300 kDa, glycoproteins like mucin > 200 kDa, for details see [20, 73, 74]. We recently showed that > 98% of all jelly-OM proteins and > 70% of jellyfish dissolved combined amino acids can be consumed by coastal microbial assemblages in less than 2 days [20]. Since bacterial transporter systems can only take up substrate smaller than 600 Da [27, 75], extracellular hydrolysis of proteins is needed prior to bacterial assimilation. Our functional analysis showed that proteins associated with protein turnover (i.e. clustered within COG category O) assigned to *Pseudoalteromonadaceae* exhibited a significantly higher abundance in the exoproteome of jelly-OM degrading consortium as compared to control treatments during late exponential growth (~ 16% of all exoproteins, Fig. 2B, Additional File 2: Table S4, S5). Detailed analysis further indicated that proteases represented ~ 10% of all proteins in the exoproteomes of jelly-OM treatments, mainly of *Pseudoalteromonadaceae* origin, suggesting a primary role of this taxa in the hydrolysis of jellyfish proteins (Fig. 3A; Additional File 2: Table S6). Collagen, one of the main constituents of the jellyfish protein pool (e.g. ~ 50% of total protein content, [76]), can be degraded only by a few types of proteases because of its special structure (triple helix, with distinctive amino acid sequence pattern Gly-Pro-Y or Gly-X-Hyp). Bacterial collagenolytic proteases mainly include metalloproteases (M9A and M9B) and serine protease (S1, S8, S53) as well as some members of the U32 family [77, 78]. The main proteases to cleave jellyfish proteins in our experiment were S8 serine proteases and M9A metalloproteases, mostly associated with *Pseudoalteromonadaceae* (Fig. 3A, Additional File 2: Table S6, Additional File 3: Fig. S2). The deseasin MCP-01 of the S8 family was shown to cleave type I, II, and IV collagens, gelatin, and fish collagen [77]. Thus, we speculate that the highly abundant proteases of the S8 family from *Pseudoalteromonadaceae* (Additional File 2: Table S6, Additional File 3: Fig. S2) in the exoproteome of the jelly-OM treatments could also cleave collagen from jellyfish, which was shown to be similar to collagen type I [79]. Microbial collagenases (and some serine proteases) were exclusively assigned to *Pseudoalteromonas* MAGs (Fig. 7; Additional File 2: Table S6, S7, S14; Additional File 3: Fig. S2), indicating that *Pseudoalteromonas*, in particular, invested energy and resources to cleave collagen from jellyfish to use its building blocks, the amino acids.

While jellyfish proteins were predominantly extracellularly hydrolyzed by *Pseudoalteromonas*, the complex jellyfish carbohydrates were mainly degraded by extracellular CAZymes affiliated to *Alteromonas* (Fig. 7; Additional File 2: S7, S14). The relative abundance of CAZymes was considerably lower as compared to proteases (~ 2% of all exoproteins, Fig. 3) in the jelly-OM treatments, in line with the fact that carbohydrates comprise only ~ 7% of total jelly-OM [31]. Glycoside hydrolases (GH) were the major CAZymes degrading jellyfish carbohydrates and were previously linked to the catabolism of mucin, i.e. the main glycoproteins constituting jellyfish mucus ([73, 80] (Additional File 3: Fig. S2). GHs are among the most abundant CAZymes in the oceanic exoproteomes [81]; yet, GHs following decay of jellyfish blooms were mostly assigned to *Alteromonas*, while GHs enriched during the senescent phase of phytoplankton blooms are predominantly of Flavobacteriales origin [82]. This implies that similar types/classes of compounds released at the decay of different bloom-forming organisms trigger metabolic responses of different bacterial taxa. Our data also reveal the main role of *Alteromonas* in the extracellular degradation of organic phosphorus compounds from jelly-OM (Fig. 7, Additional File 2: Table S14). This confirms previous results suggesting jellyfish detritus as an important source of organic and inorganic phosphorus [20, 41, 83, 84].

Even when the jelly-OM degrading community entered decay phase, 2 days after jellyfish detritus amendment, bacterial exo-metaproteomes still significantly differed from those in the control treatments (Fig. 2). At this stage, exo-metaproteomes were enriched in proteins annotated as inhibitors of proteases of *Pseudoalteromonadaceae* origin and proteins associated with inorganic ion transport/metabolism of *Alteromonadaceae*, potentially supporting substantial release of ammonium (NH_4_^+^) and PO_4_^3−^ recorded at the end of the jelly-OM degradation process (Fig. 2; Additional File 2: Table S6; [20, 29]). Altogether this suggests that chemical and metabolic fingerprints associated with jelly-OM degradation, in particular extracellular enzymes/metabolites, remain present in the water column for some time after the bloom demise.

### Niche partitioning and metabolic interactions among key jelly-OM degraders

The extracellular hydrolysis of HMW jellyfish compounds introduced a loose hydrolysis-uptake relationship and the release of public goods to the entire community, in particular by *Pseudoalteromonas* ([85, 86] and reference therein). Our analyses of the intracellular metabolism indicated a significant upregulation of the machinery associated with environmental information processing and protein/amino acids metabolic/catabolic pathways in all key jelly-OM degraders (Figs. 6 and 8, Additional File 2: Table S11, S12). Yet, we observed differences in hydrolysis, assimilation, and metabolic pathway patterns, suggesting niche partitioning among dominant MAGs constituting the jelly-OM degrading consortium (Fig. 8).

*Pseudoalteromonas* played a major role in the initial degradation of jelly-OM and specifically utilized jellyfish proteins for its growth as revealed by its set of transporters (e.g. for polar amino acids and taurine Figs. 5 and 8; [20]), by the large repertoire of endopeptidases (Additional File 2: Table S6, S13; Additional File 3: Fig. S5) and highest increase in relative abundances of protein catabolic pathways among all taxa (Figs. 6 and 8). Accordingly, *Pseudoalteromonas* represented ~ 50% of all metabolically active jelly-OM degrading bacteria and increased ~ 70-fold in absolute abundance during the jelly-OM degradation process [20]. *Alteromonas* assimilated and catabolized carbohydrates and organophosphorus compounds from jelly-OM (Figs. 5 and 6), representing ~ 30% of the metabolically active jelly-OM degrading bacteria and increased ~ 77-fold in absolute abundance as compared to ambient microbial assemblage [20]. By providing some public goods (e.g. via extracellular hydrolysis of complex carbohydrates), but at the same time exclusively exploiting a specific niche within jellyfish detritus (e.g. organophosphorus compounds), this type of interaction of *Alteromonas* with other members of jelly-OM degrading community is best described as commensalism.

*Vibrio*, however, exhibited a cheater lifestyle, i.e., exploiting public goods released by others. While the *Vibrio* MAG encoded genes for microbial collagenases (Additional File 2: Table S15), we did not detect its corresponding proteins in the metaproteomes of jelly-OM treatments (Additional File 2: Table S11-S14). At the same time, *Vibrio* activated motility, quorum sensing, chemotaxis, signal transduction, and secretory systems and was actively taking up and catabolizing peptides, amino acids, and carbohydrates, exploiting public goods released by extracellular hydrolysis of complex jelly-OM compounds conducted by *Pseudolateromonas* and *Alteromonas* (Figs. 5, 6, 7, and 8). This strategy resulted in ~ 100-fold increase in the absolute abundance of *Vibrio* (~ 12% of metabolically active bacteria) as compared to the ambient microbial assemblage (based on previous microscopy-based analysis, [20]). Besides Gammaproteobacteria, *Thalassobius* was the only Alphaproteobacterium detected within the jelly-OM degrading consortium. Transporter analysis showed that *Thalassobius* was capable of taking up peptides and amino acids (general, polar, and branched) during late exponential growth of the jelly-OM degrading community (Fig. 5, Additional File 2: Table S10). However, proteins associated with defense, filamentous growth, and quorum sensing detected in *Thalassobius* suggest antagonistic interactions with other members of the consortium resulting in suppressed growth, which could be one explanation for the observed lower relative abundance of *Thalassobius* (*Rhodobacteraceae*) during late exponential phase (Figs. 1 and 6).

The upregulation of iron scavenging was shared by all jelly-OM degraders (Additional File 2: Table S10-S12). In our dataset, the putative systems for iron uptake did not cluster within groups of TonB-dependent transporters potentially associated with uptake of DOM compounds, hence are indeed most likely allocated to iron transport [64]. One possible explanation is that extracellular enzymatic hydrolysis of polymers, the uptake of monomeric substrates and respiration can be directly controlled by iron uptake [87]. Also, bacteria might secrete iron-chelating molecules in response to phosphorus limitation [88]. During exponential growth, PO_4_^3−^ concentration was low in jelly-OM treatments, which might explain why transporters associated with phosphorus uptake were also mostly associated with the metabolically most active bacteria, *Pseudoalteromonas* and *Vibrio* (Figs. 5 and 8, microscopy-based analysis [20, 88]). Besides, the increase in abundance of siderophore biosynthesis, uptake, and metabolism proteins was most significant in *Pseudoalteromonas* and *Vibrio* during late exponential growth (Fig. 8, Additional Table S11-12). Under iron-limiting conditions, siderophores are excreted outside bacterial cell, where they strongly bind to poorly soluble iron, and are taken up via specific transporters [89]. Production of siderophores can be beneficial for the iron acquisition strategy of bacteria-colonizing jellyfish detrital particles [90]. Also, production of siderophores is linked to quorum sensing, antagonistic behaviour, and defense in some bacteria [91–93]. This could explain the predominance of *Pseudoalteromonas* and *Vibrio* over other bacterial populations in the jelly-OM degrading community.

Once most of the labile jelly-OM is depleted (within ~ 1.5 days) the microbial metabolic response decreases, evident from only ~ 4% of proteins being differentially abundant as compared to the control treatments (Figs. 4 and 6; Additional File 2: Table S9, Additional File 3: Fig. S3, S4) and the jelly-OM degrading community enters the decay/senescent phase of their growth [20]. We observed a rapid decline of *Pseudoalteromonas* and *Vibrio* populations (conforming to the phenomena of tragedy of commons; [94]). The left-overs, end-, and by-products of the first stage of bacterial jelly-OM degradation and/or even compounds released during cell burst of *Pseudoalteromonas* and *Vibrio* populations created an environment which supported growth of a more diverse community (Fig. 1, Additional File 2: Table S3). Growth of *Alteromonas* (~ 50% of respiring senescent population, [20]) and filamentous *Rhodobacteraceae Thalassobius* (Fig. 1, microscopy images of filamentous bacteria, [20]) was maintained by catabolism of proteins, amino acids, and nucleobases (Figs. 2B and 6A,B). It is likely that these metabolic processes fueled the accumulation of different compounds of the dissolved organic nitrogen pool during decay phase [20].

## Conclusions

By combining metagenomics with a metaproteomics approaches, we provide first insights into the metabolic network operated by jelly-OM degraders. Our analysis revealed that the decay of jellyfish blooms triggers a rapid re-shuffling of the taxonomic composition and functional profile of the ambient bacterial community, which is substantially different to the one previously associated with decaying phytoplankton blooms. Our research thus highlights the importance of considering seasonal and/or episodic blooms of gelatinous zooplankton as important force perturbating the metabolism of ambient microbial communities. The specific metabolic fingerprint, in particular the enrichment in extracellular collagenolytic proteases by *Pseudoalteromonas* potentially acts as a tool of bacterial defense and exhibit an antagonistic activity as well as acts as potential virulence factors for humans and marine organisms’ disease. This might represent an important aspect of decaying jellyfish blooms, with potential implications for marine ecosystem functioning and its services [95]. Further functional characterization of marine bacterial collagenolytic proteases will provide important information on the turnover of collagen in the marine environment with biotechnological potential beyond its ecological context. The predominance of collagenases following the decay of jellyfish blooms represents a unique protease fingerprint, different from the profile of proteases of the global ocean [81, 96].

Our data provide lines of evidence pointing to the key role of *Pseudoalteromonas, Alteromonas,* and *Vibrio* during the initial jelly-OM degradation and the potential importance of *Alteromonas* and *Thalassobius* during the decay phase of jellyfish blooms. In addition, we also provide novel insights into niche partitioning within bacterial communities when facing a complex pool of OM, which supports complex interactions among providers of public good and co-perators (*Pseudoalteromonas*, *Alteromonas*), cheaters (*Vibrio*), and antagonistic behaviour (towards *Thalassobius*) within the degrading bacterial consortium. We show that the bacterial community needs to operate a complex metabolic network in a temporal cascade of biochemical reactions to degrade jellyfish-bloom-specific compounds. Furthermore, specific chemical and metabolic fingerprints associated with jelly-OM degradation, mainly extracellular enzymes and metabolites, remain present in the water for some time after the bloom demise, with implications for the biogeochemistry of the affected system.

However, to approach ecological reality, in situ experiments should be conducted in the future, which is challenging due to the patchiness of jellyfish blooms and current inability to track bloom development and population dynamics remotely. Also, the use of fresh jellyfish carcasses instead of dry jellyfish detritus should be considered and the role of the jellyfish-native microbiota in the degradation process of its host should be investigated. Nevertheless, our results fill an important knowledge gap in our understanding of jellyfish-microbe interactions, representing an important step towards a more accurate integration of jellyfish-derived OM into biogeochemical models.

## Supplementary Information

**Additional file 1.**

**Additional file 2: Table S1.** Number of proteins recruited in different fractions (Endo-protemes, Exo-proteomes: High Molecular Weight fraction (ExoH), Low Molecular Weigth Fraction (ExoL)) from the coastal assemblage, at the late exponential phase of bacterial growth and during decay phase of bacterial community growth in jellyfish (biological replicates J1, J2 and J3) and control (biological replicates C1, C2, C3) treatment. Pre-filtration step ≥1 unique peptides and ≥2peptides was applied.** Table S2.** Statistical analysis of taxonomic origin of proteins of meta- and exo-proteomes from jelly-OM and control treatments on super kingdom, class, order and family level. Provided as separate excel/csv files.** Table S3.** α-diversity of genes and associated proteins of meta- and exo-proteomes from jellyOM and control treatments based on Shannon diversity index. Statistics are based on Wilcoxon test, *P < 0.05, **P < 0.01, ***P < 0.001, ****P < 0.0001, ns, not significant. Provided as separate excel/csv files.** Table S4.** Relative abundance of significantly enriched COG categories in jelly-OM vs control treatments. COG representing at least 1% of all detected proteins in at least one sample during different growth phase of bacterial growth are listed. Provided as separate excel/csv files.** Table S5.** Taxonomic origin of proteins associated with specific COG category, with statistical analysis. Provided as separate excel/csv files.** Table S6.** Relative abundance of total proteases, families of proteases and inhibitors of proteases significantly enriched in jelly-OM vs control treatments during different growth phase of bacterial growth and their taxonomic origin. Provided as separate excel/csv files.** Table S7.** Results of signal peptides analysis. Provided as separate excel/csv files.** Table S8.** Relative abundance of total carbohydrate-active enzymes, families of CAZymes significantly enriched in jelly-OM vs control treatments during different growth phase of bacterial growth and their taxonomic origin. Provided as separate excel/csv files.** Table S9.** Differential expressed genes and corresponding proteins (log2FC, q-values) associated with each key MAG of jelly-OM degrading community. Provided as separate excel/csv files.** Table S10.** Relative abundance of individual genes and corresponding proteins associated with transport per MAG during late exponential and decay phase of bacterial community growth in jelly-OM and control treatments. Provided as separate excel/csv files.** Table S11.** GO terms annotations of upregulated genes and associated proteins during different phase of bacterial community growth (log2FC, q-values). Shared among all key jelly-OM degraders. Unique for Gamma- vs Alpha- proteobacteria. Unique for Pseudoalteromonas, Alteromonas, Vibrio and Thalassobius. Provided as separate excel/csv files.** Table S12.** KEGG annotated individual genes (log2FC, q-values) per MAG during different phase of bacterial community growth. Provided as separate excel/csv files.** Table S13.** Relative abundance of endo- and exo-proteases per MAG during late exponential and decay phase of bacterial community growth in jelly-OM and control treatments (supplementary to manuscript Figure S5). Provided as separate excel/csv files.** Table S14.** Relative abundance of exo-proteins associated with each MAG during different growth phases of the bacterial community in the jelly-OM and control treatments. Provided as separate excel/csv files.** Table S15.** All KEGG annotated genes and associated proteins per each key MAG of jellyOM degrading consortia.

**Additional file 3: Figure S1.** Taxonomic origin of proteins at A) superkingdom, B) class, C) order and D) family level. Higher taxonomic level was assigned when taxa could not be classified down to class/order/family level. Note: in y axes 1 equals 100%.** Figure S2.** Relative abundance of protease (A) and CAZyme (B) subfamilies significantly enriched in meta- and exo-proteomes from jelly-OM as compared to control treatments. Note: in y-axes 1 equals 100%.** Figure S3.** Supplementary to Figure 4: High-resolution heatmaps and Venn diagrams of differentially abundant proteins for each key MAG during different stages of the jelly-OM degradation process. Top panels: Gene: the total gene profile of each MAG: genes encoding peptidases are in yellow, genes encoding transporters in blue, the rest is shown in grey. The false discovery rate (FDR) of log2 transformed fold change (Log2FC) of all proteins per MAG after jelly-OM amendments during the late exponential (E2_FDR) and decay (E1_FDR) phase, FDR <0.05 is highlighted using (*). Log2FC of individual proteins during late exponential (black dots) and decay phase (red dots) is presented. In the heatmap, jellyfish treatments are highlighted as bars in blue, in orange the control treatments, late exponential phase in dark blue (E2) and decay phase in green (E1). Bottom panels: Numbers of differentially abundant proteins for which relative abundance increased (up) or decreased (down) exclusively during late exponential (E2) and/or decay phase (E1) and/or throughout the jelly-OM degradation process are also presented as Venn diagrams.** Figure S4**. A.) GO-annotated and B.) KEGG-annotated differentially abundant proteins (enrichment as log2FC and significance as p-value or FDR) per each MAG during different stage of jelly-OM degradation process. *Pseudoalteromonas*: bin22 and bin24; *Alteromonas*: bin29; *Vibrio*: bin3; *Thalassobius*: bin51. Circles: proteins related to biological processes, Triangles: proteins related to cellular components, Squares: proteins related to molecular function. Blue: proteins up-regulated in jelly-OM (as compared to control) treatments. Yellow: proteins down-regulated in jelly-OM (as compared to control) treatments. Grey: proteins with no significant difference in expression between jelly-OM and control treatments. E2: late exponential growth phase of jelly-OM degrading community; E1: decay phase of jelly-OM degrading community.

## Data Availability

All data needed to evaluate the conclusions of the article are present in the article and/or the Supplementary Material. Raw reads of all metagenomic DNA libraries are deposited at NCBI under the accession number PRJNA633735. Metagenome assemblies are available at the following link (https://figshare.com/s/bc4c0fb59e3d0d3e8aeb). The mass spectrometry proteomics data have been deposited to the ProteomeXchange Consortium [51] via the PRIDE [52] partner repository with the dataset identifier PXD036989. Additional data related to this article may be requested from the corresponding author.
