# Supplementary material for: Jellyfish detritus supports niche partitioning and metabolic interactions among pelagic marine bacteria
Source: Microbiome. 2023 Jul 21;11:156. doi: 10.1186/s40168-023-01598-8 (PMC10360251; doi:10.1186/s40168-023-01598-8)
Supplement: Supplementary file 2 — Additional file 1. [file 40168_2023_1598_MOESM1_ESM.pdf]

Additional file 1 to Tinta et al. Jellyfish detritus supports niche partitioning and metabolic interactions among marine bacteria

Detailed Methodology: description of sample preparation, processing and analytical pipeline related to metagenomes and proteome analysis

Metagenomes

Sample preparation and sequencing

Bacterial biomass was collected onto 0.22 µm polyether sulfone membrane filters (PALL Inc.) by filtering 2 L of the bacterial inoculum before the start of the experiments and 2 L of each of the triplicate control treatments and 0.5 L of each of the triplicate jellyfish treatments at 32h when the peak in bacterial abundance was reached in the jellyfish treatment. Total nucleic acids were extracted following the protocol published elsewhere [1] with some modifications for the extraction from filters. For details of the extraction protocol please see Supplementary Material S Info 5 and [2]. We have sequenced the metagenome of the coastal microbiome (by pooling DNA extracted from the bacterial inoculum used to set up each of the two experiments) and of the communities from jelly-OM and the control treatments (by pooling DNA extracted from each, the triplicate jelly-OM and the control flasks). All three metagenomic DNA libraries were constructed individually (Westburg kit, enzymatic shearing) and sequenced on one lane of the HiSeqV4 Illumina platform at the Vienna Biocenter Core Facilities.

Data analysis pipeline

Paired-end reads were assembled from each metagenome with MEGAHIT v.1.1.1 with default setting[3]. Gene prediction was performed with Prodigal under metagenomic mode (-p meta) [4]. Functional annotation for predicted gene was performed by searching against EggNOG database v5.0 using eggNOG-mapper v2 [5]. The predicted gene was further annotated against

dbCAN database [6, 7] and MEROPs database [8] for CAZyme and peptidase identification, respectively. Taxonomic affiliation of the predicted genes was identified using the lowest common ancestor algorithm adapted from DIAMOND 0.8.36 blast [9]by searching against the NCBI non-redundant (NR) database [10]. The top 10% hits with an e-value <1 x 10 -5 were used for phylogenetic assignment (–top 10). Reads recruitment was performed using the BWA algorithm (bwa mem) (0.7.16a) [11] to evaluate the gene abundance. The gene abundance was estimated by the number of mapped reads and normalized as follows: RPM (mapped reads per million) = 106 x (mapped reads/gene length)/sum of (mapped reads/gene length). For MAG construction, paired-end reads from each metagenome were pooled for co-assembled using MEGAHIT v.1.1.1. Two separate automatic binning algorithms, MaxBin and MetaBAT (2.15), were employed for binning [12, 13] . The generated genomic bins were de-replicated and refined with Metawrap (bin_refinement) [14]. Bins with >70% completeness and <10% contamination were kept for downstream analysis (-c 70, x 10). To determine the abundances of the bins across samples, short reads from each metagenome were mapped to the bins using the Metawrap function “quant_bins.”

Proteomes

Protein extraction from filters: Filters were ground into small pieces with a sterile metal spatula after submerging the tubes with the filters into liquid nitrogen. Filter pieces were resuspended in lysis buffer (100mM Tris-HCl pH 7.4, 1% SDS, 150mM NaCl, 1mM DTT, 10mM EDTA) and cells were lysed with five freeze-and-thaw cycles. After centrifugation (20,000 g at 4°C for 25 min) the supernatant was transferred into a tube and proteins were co-precipitated with 0.015% deoxycholate and 6% trichloroacetic acid (TCA) on ice for 1h and washed once with ice-cold acetone according to the protocol of [15]. Dried protein pellets were resuspended in 50 µL of 8 M urea containing 4% SDS and protein concentrations were measured with the bicinchoninic acid (BCA) assay using BSA as a standard. Between 10-50 µg of protein were

subjected to denaturing polyacrylamide gel-electrophoresis (SDS-PAGE), stained with Coomassie staining solution and de-stained in 40% (v/v) methanol containing 2% (v/v) acetic acid as described in [16]. Gel bands were excised without fractionating the bands, cut into 1 x 1 mm pieces on a sterile glass plate and transferred into protein-low binding tubes (Eppendorf). Gel pieces were de-stained with 25 mM triethylammonium bicarbonate buffer containing 50% acetonitrile at room temperature overnight, followed by 100% acetonitrile incubation and dried. Next, cysteines were reduced and alkylated with 10 mM DTT and 55 mM iodoacetamide (IAA), respectively, as described by [17]prior to overnight trypsin digestion at 37°C according to [16]. Trypsin digestion was terminated by adding trifluoroacetic acid (TFA) to the samples (1% final concentration in 50% acetonitrile). Samples were desalted using Pierce C18 Tips (Thermo Scientific) according to manufacturer's instructions. Prior the LC MS/MS analyses, samples were dissolved in 0.1% formic acid and 2% acetonitrile and transferred into microinserts sealed with aluminium caps. Beforehand, peptides were quantified using Pierce quantitative fluorometric peptide assay (Thermo Scientific) according to manufacturer's protocol. The concentration of peptides ranged from 22±11 ng μL^{-1} to 49±11 ng μL^{-1} in control and jellyfish treatments, respectively.

Protein extraction from filtrate: Proteins were precipitated with 9 volumes of 96% EtOH at -20°C overnight for purification. Protein pellets were resuspended with 50 mM TEAB buffer (Sigma) and quantified using Pierce 660nm Protein Assay Reagent (ThermoFisher). Thereafter, cysteines were reduced and alkylated as described above, followed by another protein precipitation with 9 volumes of 96% EtOH at -20°C overnight. Again, pellets were resuspended in 50 mM TEAB, followed by overnight in-solution trypsin (Roche) digestion (1:100, w/w) at 37°C. TFA was added to the samples at 1% final concentration to terminate trypsin digestion. Samples were desalted using Pierce C18 Tips (Thermo Scientific) according to the manufacturer's protocol. Prior the LC MS/MS analyses, digested peptides were dissolved

in 0.1% formic acid and 2% acetonitrile and transferred into micro-inserts sealed with aluminium caps. Before the run, the concentration of peptides was measured using Pierce Quantitative fluorometric peptide assay (Thermo Scientific). Concentration of peptides ranged from 18±5 ng μL^{-1} to 55±30 ng μL^{-1} for control and jellyfish treatments, respectively.

LC-MS/MS Analysis and Peptide Identification

Five μL of desalinated peptides were analyzed on Orbitrap Elite mass spectrometer (Thermo Fisher Scientific) as previously described [18]. The MS/MS spectra from each proteomic sample was searched using SEQUEST engines against predicted gene category and validated with Percolator in Proteome Discoverer 2.1 (Thermo Fisher Scientific) by employing the settings described in [19]. Briefly, to reduce the probability of false peptide identification, the target–decoy approach was used and results <1% FDR at the peptide level were kept [20]. A minimum of two peptides and one unique peptide was required for protein identification. Protein quantification was conducted with a chromatographic peak area-based label-free quantitative method [21].

References

1. Angel R. Total Nucleic Acid Extraction from Soil. Protoc Exch. 2012. https://doi.org/10.1038/protex.2012.046.

2. Tinta T, Zhao Z, Escobar A, Klun K, Bayer B, Amano C, et al. Microbial Processing of Jellyfish Detritus in the Ocean. Front Microbiol. 2020;11.

3. Li D, Luo R, Liu C-M, Leung C-M, Ting H-F, Sadakane K, et al. MEGAHIT v1.0: A fast and scalable metagenome assembler driven by advanced methodologies and community practices. Methods. 2016;102:3–11.

4. Hyatt D, Chen G-L, Locascio PF, Land ML, Larimer FW, Hauser LJ. Prodigal: prokaryotic gene recognition and translation initiation site identification. 2010.

5. Huerta-Cepas J, Szklarczyk D, Heller D, Hernández-Plaza A, Forslund SK, Cook H, et al. EggNOG 5.0: A hierarchical, functionally and phylogenetically annotated orthology resource based on 5090 organisms and 2502 viruses. Nucleic Acids Res. 2019;47:D309–14.

6. Yin Y, Mao X, Yang J, Chen X, Mao F, Xu Y. dbCAN: a web resource for automated carbohydrate-active enzyme annotation. Nucleic Acids Res. 2012;40:W445–51.

7. Cantarel BL, Coutinho PM, Rancurel C, Bernard T, Lombard V, Henrissat B. The Carbohydrate-Active EnZymes database (CAZy): an expert resource for Glycogenomics. Nucleic Acids Res. 2009;37 suppl_1:D233–8.

8. Rawlings ND, Barrett AJ, Thomas PD, Huang X, Bateman A, Finn RD. The MEROPS database of proteolytic enzymes, their substrates and inhibitors in 2017 and a comparison with peptidases in the PANTHER database. Nucleic Acids Res. 2018;46:D624–32.

9. Buchfink B, Xie C, Huson DH. Fast and sensitive protein alignment using DIAMOND. Nat Methods. 2015;12:59–60.

10. Sayers EW, Beck J, Bolton EE, Bourexis D, Brister JR, Canese K, et al. Database resources of the National Center for Biotechnology Information. Nucleic Acids Res. 2021;49:D10–7.

11. Li H, Durbin R. Fast and accurate long-read alignment with Burrows–Wheeler transform. Bioinformatics. 2010;26:589–95.

12. Wu Y-W, Tang Y-H, Tringe SG, Simmons BA, Singer SW. MaxBin: an automated binning method to recover individual genomes from metagenomes using an expectation-maximization algorithm. Microbiome. 2014;2:26.

13. Kang DD, Froula J, Egan R, Wang Z. MetaBAT, an efficient tool for accurately reconstructing single genomes from complex microbial communities. PeerJ. 2015;2015.

14. Uritskiy G v, DiRuggiero J, Taylor J. MetaWRAP—a flexible pipeline for genome-resolved metagenomic data analysis. Microbiome. 2018;6:158.

15. Bensadoun A, Weinstein D. Assay of proteins in the presence of interfering materials. Anal Biochem. 1976;70:241–50.

16. Valledor L, Weckwerth W. An Improved Detergent-Compatible Gel-Fractionation LC-LTQ-Orbitrap-MS Workflow for Plant and Microbial Proteomics. In: Jorrin-Novo J v, Komatsu S, Weckwerth W, Wienkoop S, editors. Plant Proteomics: Methods and Protocols. Totowa, NJ: Humana Press; 2014. p. 347–58.

17. Shevchenko A, Tomas H, Havli J, Olsen J v, Mann M. In-gel digestion for mass spectrometric characterization of proteins and proteomes. Nat Protoc. 2006;1:2856–60.

18. Bayer B, Pelikan C, Bittner MJ, Reinthaler T, Könneke M, Herndl GJ, et al. Proteomic Response of Three Marine Ammonia-Oxidizing Archaea to Hydrogen Peroxide and Their Metabolic Interactions with a Heterotrophic Alphaproteobacterium. mSystems. 2019;4:e00181-19.

19. Hansen HP, Koroleff F. Determination of nutrients. In: Methods of Seawater Analysis. 1999. p. 159–228.

20. Elias JE, Gygi SP. Target-decoy search strategy for increased confidence in large-scale protein identifications by mass spectrometry. Nat Methods. 2007;4:207–14.

21. Zhang Y, Wen Z, Washburn MP, Florens L. Improving Label-Free Quantitative Proteomics Strategies by Distributing Shared Peptides and Stabilizing Variance. Anal Chem. 2015;87:4749–56.
