# Supplementary material for: Jellyfish detritus supports niche partitioning and metabolic interactions among pelagic marine bacteria
Source: Microbiome. 2023 Jul 21;11:156. doi: 10.1186/s40168-023-01598-8 (PMC10360251; doi:10.1186/s40168-023-01598-8)
Supplement: Supplementary file 3 — Additional file 2: Table S1. Number of proteins recruited in different fractions (Endo-protemes, Exo-proteomes: High Molecular Weight fraction (ExoH), Low Molecular Weigth Fraction (ExoL)) from the coastal assemblage, at the late exponential phase of bacterial growth and during decay phase of bacterial community growth in jellyfish (biological replicates J1, J2 and J3) and control (biological replicates C1, C2, C3) treatment. Pre-filtration step ≥1 unique peptides and ≥2peptides was applied. Table S2. Statistical analysis of taxonomic origin of proteins of meta- and exo-proteomes from jelly-OM and control treatments on super kingdom, class, order and family level. Provided as separate excel/csv files. Table S3. α-diversity of genes and associated proteins of meta- and exo-proteomes from jellyOM and control treatments based on Shannon diversity index. Statistics are based on Wilcoxon test, *P < 0.05, **P < 0.01, ***P < 0.001, ****P < 0.0001, ns, not significant. Provided as separate excel/csv files. Table S4. Relative abundance of significantly enriched COG categories in jelly-OM vs control treatments. COG representing at least 1% of all detected proteins in at least one sample during different growth phase of bacterial growth are listed. Provided as separate excel/csv files. Table S5. Taxonomic origin of proteins associated with specific COG category, with statistical analysis. Provided as separate excel/csv files. Table S6. Relative abundance of total proteases, families of proteases and inhibitors of proteases significantly enriched in jelly-OM vs control treatments during different growth phase of bacterial growth and their taxonomic origin. Provided as separate excel/csv files. Table S7. Results of signal peptides analysis. Provided as separate excel/csv files. Table S8. Relative abundance of total carbohydrate-active enzymes, families of CAZymes significantly enriched in jelly-OM vs control treatments during different growth phase of bacterial growth and their [file 40168_2023_1598_MOESM2_ESM.zip › Additional_File_2.pdf]

Additional file 2 to Tinta et al. Jellyfish detritus supports niche partitioning and metabolic interactions among marine bacteria

Supplementary Tables: list of supplementary tables, pdf. Supplementary tables provided as in separate ZIP folder, within which organized in subfolders with csv files.

Table S1: Number of proteins recruited in different fractions (Endo-protemes, Exo-proteomes: High Molecular Weight fraction (ExoH), Low Molecular Weigth Fraction (ExoL)) from the coastal assemblage, at the late exponential phase of bacterial growth and during decay phase of bacterial community growth in jellyfish (biological replicates J1, J2 and J3) and control (biological replicates C1, C2, C3) treatment. Pre-filtration step ≥1 unique peptides and ≥2peptides was applied.

		Late exponential phase of bacterial growth						Decay phase of bacterial growth					
	Coastal	C1	C2	C3	J1	J2	J3	C1	C2	C3	J1	J2	J3
Endo	10661	10075	11502	11645	10613	10782	10809	8088	10248	10775	11259	10967	11142
ExoH	368	1821	1858	1684	5955	5397	5506	622	865	608	6302	4442	4434
ExoL	445	558	508	381	2353	1803	2252	659	1138	610	1236	1238	1600

Table S2: Statistical analysis of taxonomic origin of proteins of meta- and exo-proteomes from jelly-OM and control treatments on super kingdom, class, order and family level. Provided as separate excel/csv files.

Table S3: α-diversity of genes and associated proteins of meta- and exo-proteomes from jelly-OM and control treatments based on Shannon diversity index. Statistics are based on Wilcoxon test, *$P < 0.05$, **$P < 0.01$, ***$P < 0.001$, ****$P < 0.0001$, ns, not significant. Provided as separate excel/csv files.

Table S4: Relative abundance of significantly enriched COG categories in jelly-OM vs control treatments. COG representing at least 1% of all detected proteins in at least one sample during different growth phase of bacterial growth are listed. Provided as separate excel/csv files.

Table S5: Taxonomic origin of proteins associated with specific COG category, with statistical analysis. Provided as separate excel/csv files.

Table S6: **A**) Relative abundance of total proteases, families of proteases and inhibitors of proteases significantly enriched in jelly-OM vs control treatments during different growth phase of bacterial growth and their taxonomic origin. Provided as separate excel/csv files.

Table S7: Results of signal peptides analysis. Provided as separate excel/csv files.

Table S8: Relative abundance of total carbohydrate-active enzymes, families of CAZymes significantly enriched in jelly-OM vs control treatments during different growth phase of bacterial growth and their taxonomic origin. Provided as separate excel/csv files

Table S9: Differential expressed genes and corresponding proteins (log2FC, q-values) associated with each key MAG of jelly-OM degrading community. Provided as separate excel/csv files

Table S10: Relative abundance of individual genes and corresponding proteins associated with transport per MAG during late exponential and decay phase of bacterial community growth in jelly-OM and control treatments. Provided as separate excel/csv files

Table S11: GO terms annotations of upregulated genes and associated proteins during different phase of bacterial community growth (log2FC, q-values). Shared among all key jelly-OM degraders. Unique for Gamma- vs Alpha- proteobacteria. Unique for *Pseudoalteromonas, Alteromonas*, *Vibrio* and *Thalassobius*. Provided as separate excel/csv files

Table S12: KEGG annotated individual genes (log2FC, q-values) per MAG during different phase of bacterial community growth. Provided as separate excel/csv files

Table S13: Relative abundance of endo- and exo-proteases per MAG during late exponential and decay phase of bacterial community growth in jelly-OM and control treatments (supplementary to manuscript Figure S5). Provided as separate excel/csv files

Table S14: Relative abundance of exo-proteins associated with each MAG during different growth phases of the bacterial community in the jelly-OM and control treatments. Provided as separate excel/csv files

Table S15: All KEGG annotated genes and associated proteins per each key MAG of jelly-OM degrading consortia
