# Supplementary material for: Jellyfish detritus supports niche partitioning and metabolic interactions among pelagic marine bacteria
Source: Microbiome. 2023 Jul 21;11:156. doi: 10.1186/s40168-023-01598-8 (PMC10360251; doi:10.1186/s40168-023-01598-8)
Supplement: Supplementary file 4 — Additional file 3: Figure S1. Taxonomic origin of proteins at A) superkingdom, B) class, C) order and D) family level. Higher taxonomic level was assigned when taxa could not be classified down to class/order/family level. Note: in y axes 1 equals 100%. Figure S2. Relative abundance of protease (A) and CAZyme (B) subfamilies significantly enriched in meta- and exo-proteomes from jelly-OM as compared to control treatments. Note: in y-axes 1 equals 100%. Figure S3. Supplementary to Figure 4: High-resolution heatmaps and Venn diagrams of differentially abundant proteins for each key MAG during different stages of the jelly-OM degradation process. Top panels: Gene: the total gene profile of each MAG: genes encoding peptidases are in yellow, genes encoding transporters in blue, the rest is shown in grey. The false discovery rate (FDR) of log2 transformed fold change (Log2FC) of all proteins per MAG after jelly-OM amendments during the late exponential (E2_FDR) and decay (E1_FDR) phase, FDR <0.05 is highlighted using (*). Log2FC of individual proteins during late exponential (black dots) and decay phase (red dots) is presented. In the heatmap, jellyfish treatments are highlighted as bars in blue, in orange the control treatments, late exponential phase in dark blue (E2) and decay phase in green (E1). Bottom panels: Numbers of differentially abundant proteins for which relative abundance increased (up) or decreased (down) exclusively during late exponential (E2) and/or decay phase (E1) and/or throughout the jelly-OM degradation process are also presented as Venn diagrams. Figure S4. A.) GO-annotated and B.) KEGG-annotated differentially abundant proteins (enrichment as log2FC and significance as p-value or FDR) per each MAG during different stage of jelly-OM degradation process. Pseudoalteromonas: bin22 and bin24; Alteromonas: bin29; Vibrio: bin3; Thalassobius: bin51. Circles: proteins related to biological processes, Triangles: proteins related to cellular components, Squ [file 40168_2023_1598_MOESM3_ESM.pdf]

**Additional file 3** to Tinta et al. Jellyfish detritus supports niche partitioning and metabolic interactions among marine bacteria

## Supplementary Figures

**Figure S1**: Taxonomic origin of proteins at **A**) superkingdom, **B**) class, **C**) order and **D**) family level. Higher taxonomic level was assigned when taxa could not be classified down to class/order/family level. Note: in y axes 1 equals 100%.

### A. Superkingdom

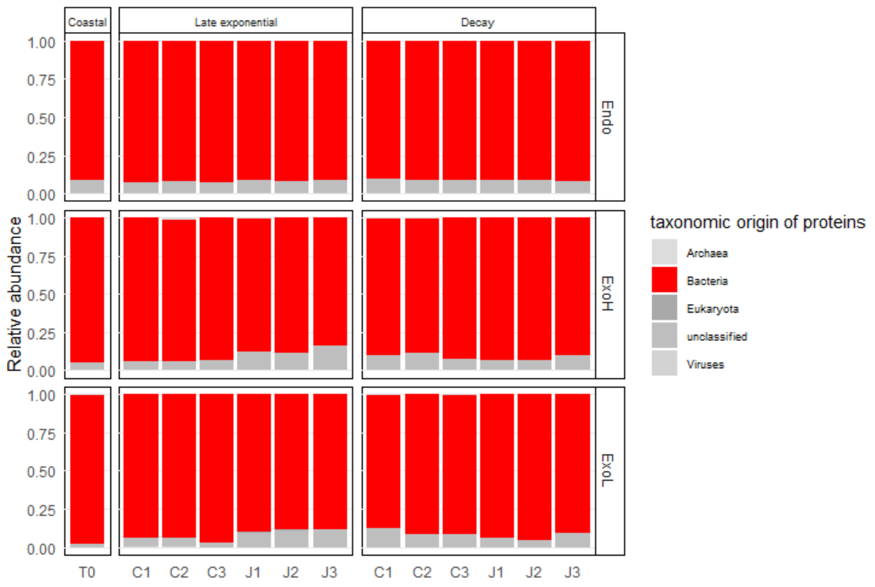


### B. Class

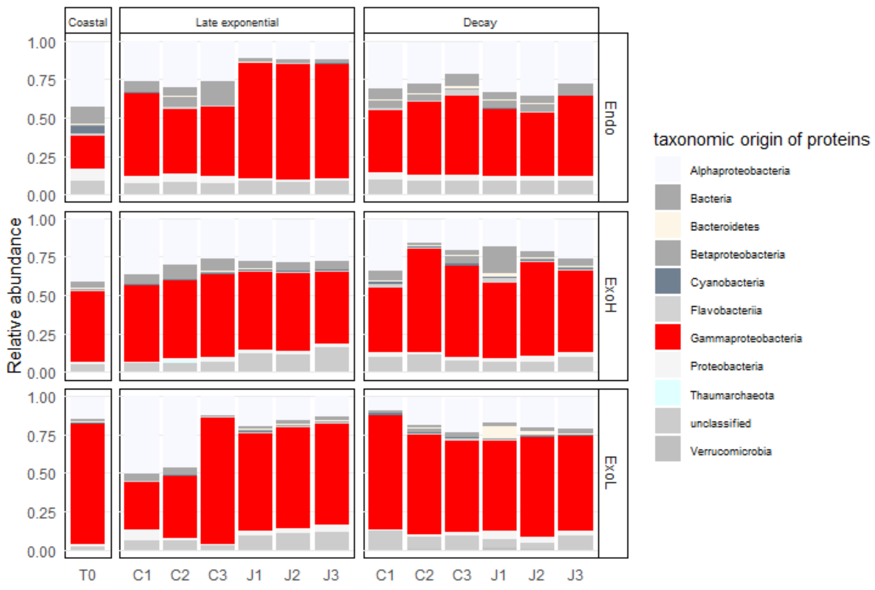

## C. Order

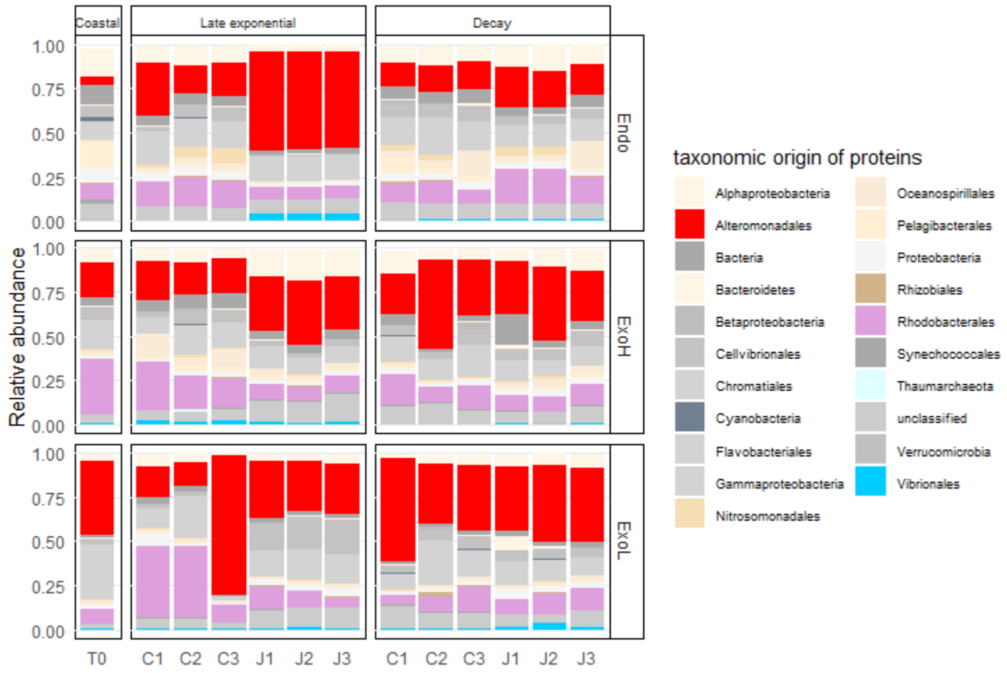


## D. Family

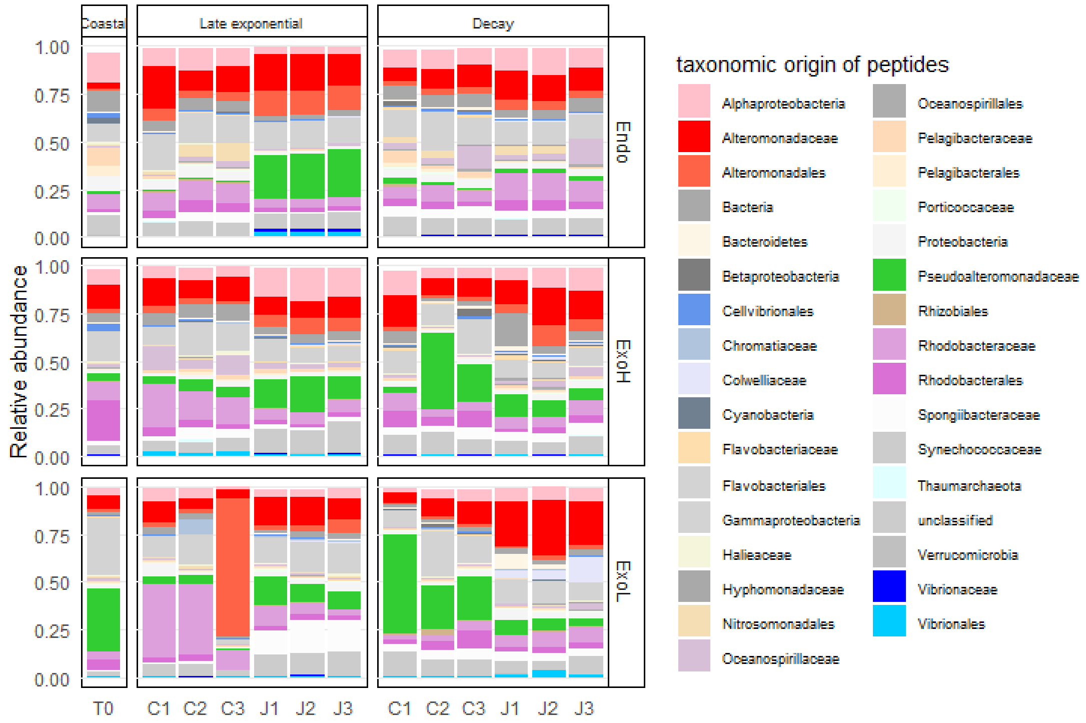

**Figure S2**: Relative abundance of protease (A) and CAZyme (B) subfamilies significantly enriched in meta- and exo-proteomes from jelly-OM as compared to control treatments. Note: in y-axes 1 equals 100%.

**A.1 Subfamilies of protease.** C – cysteine peptidase, M – metallo peptidase, N – aspargine peptidase, S – serine peptidase, T – threonine peptidases, U – unknown. Consecutive number is subfamily identifier.

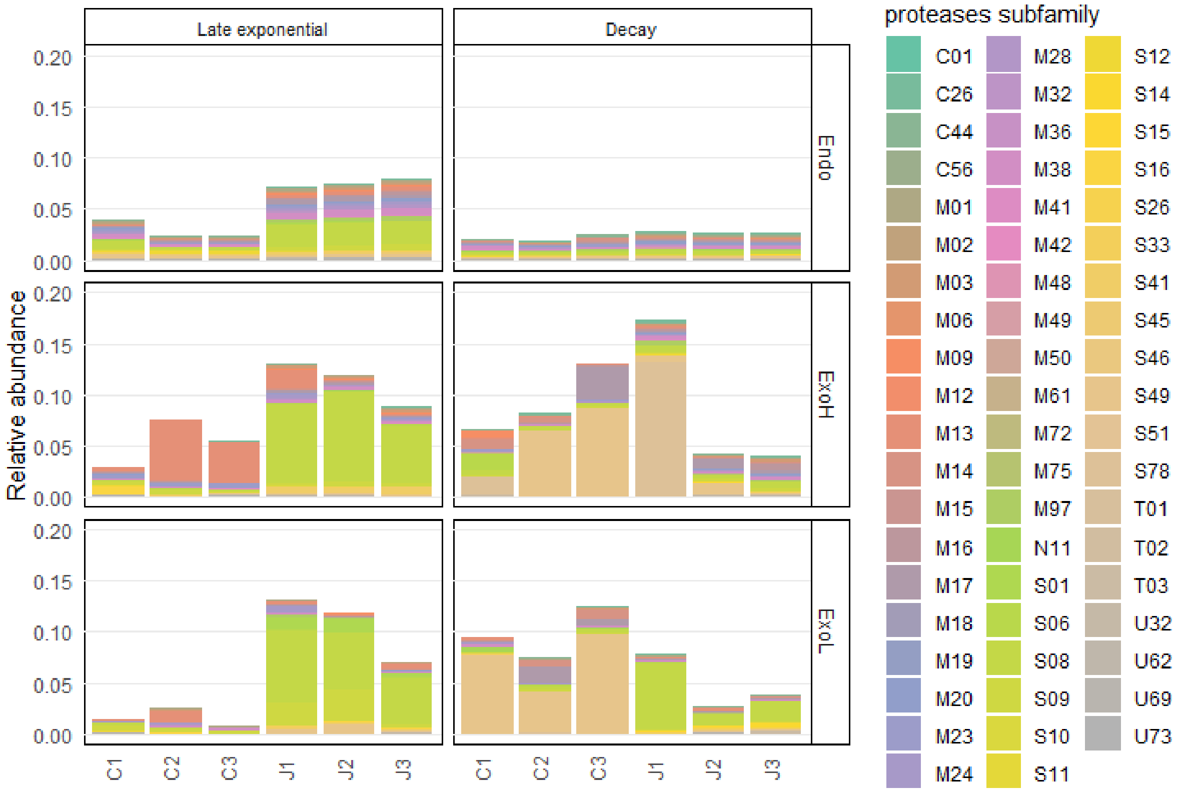

## A.2 Taxonomic origin of S08 subfamily

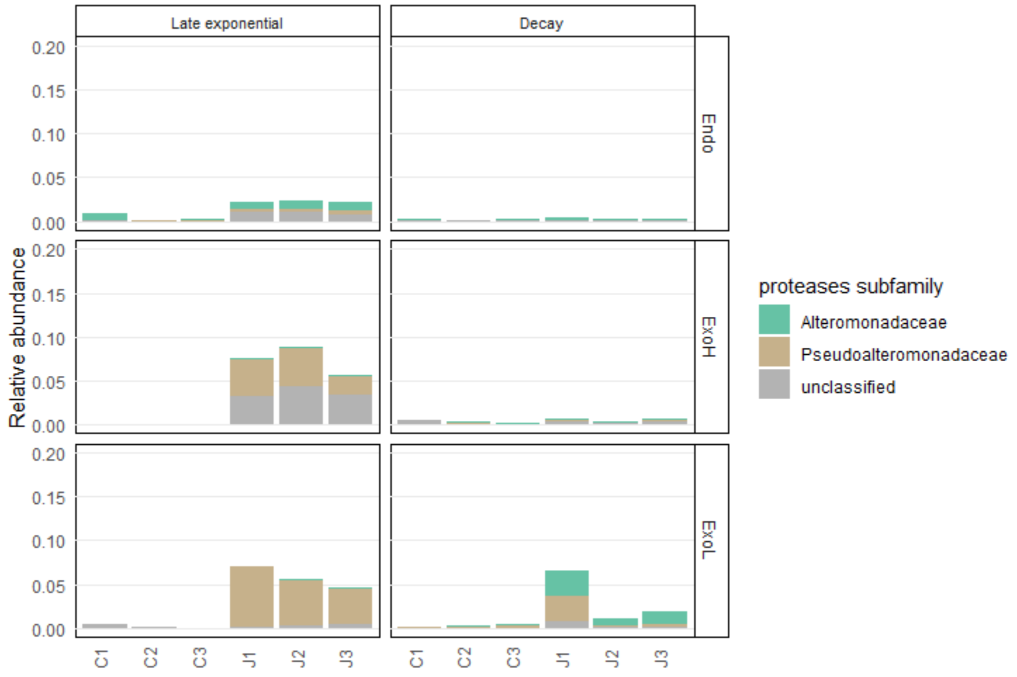


## A.3 Taxonomic origin of M9A subfamily

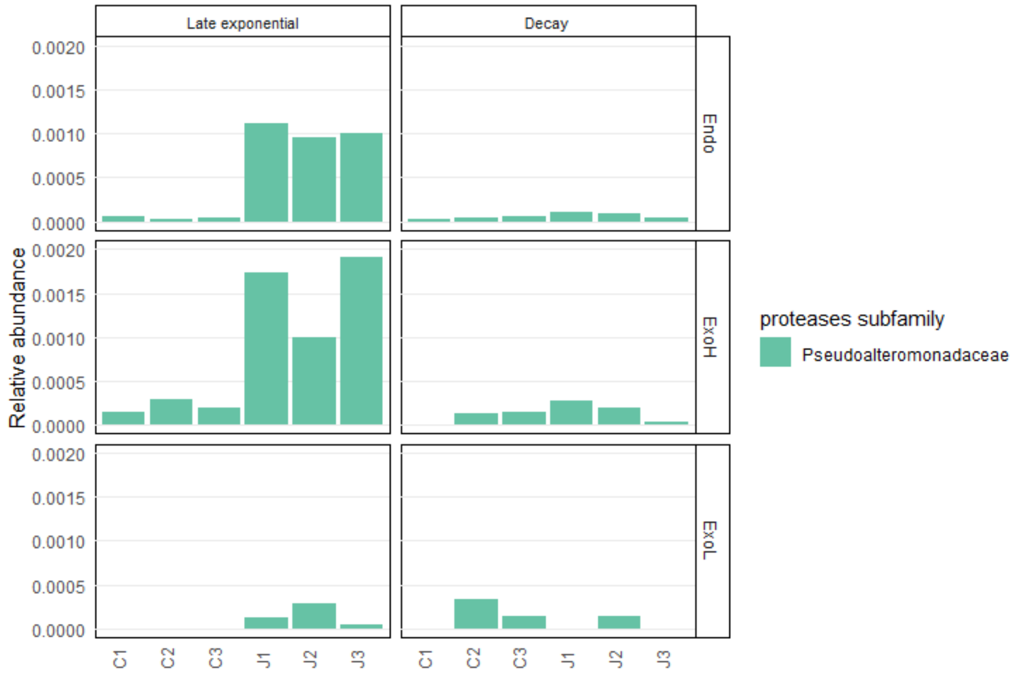

**B. CAZyme subfamilies**: AA – Auxiliary Activities, CB – Carbohydrate-Biding Modules, CE – Carbohydrate Esterases, GH – Glycoside Hydrolases, GT – Glycosyl Transfersases, PL – Polysaccharide lyases, SL – Unknown. Note: in y-axes 1 equals 100%.

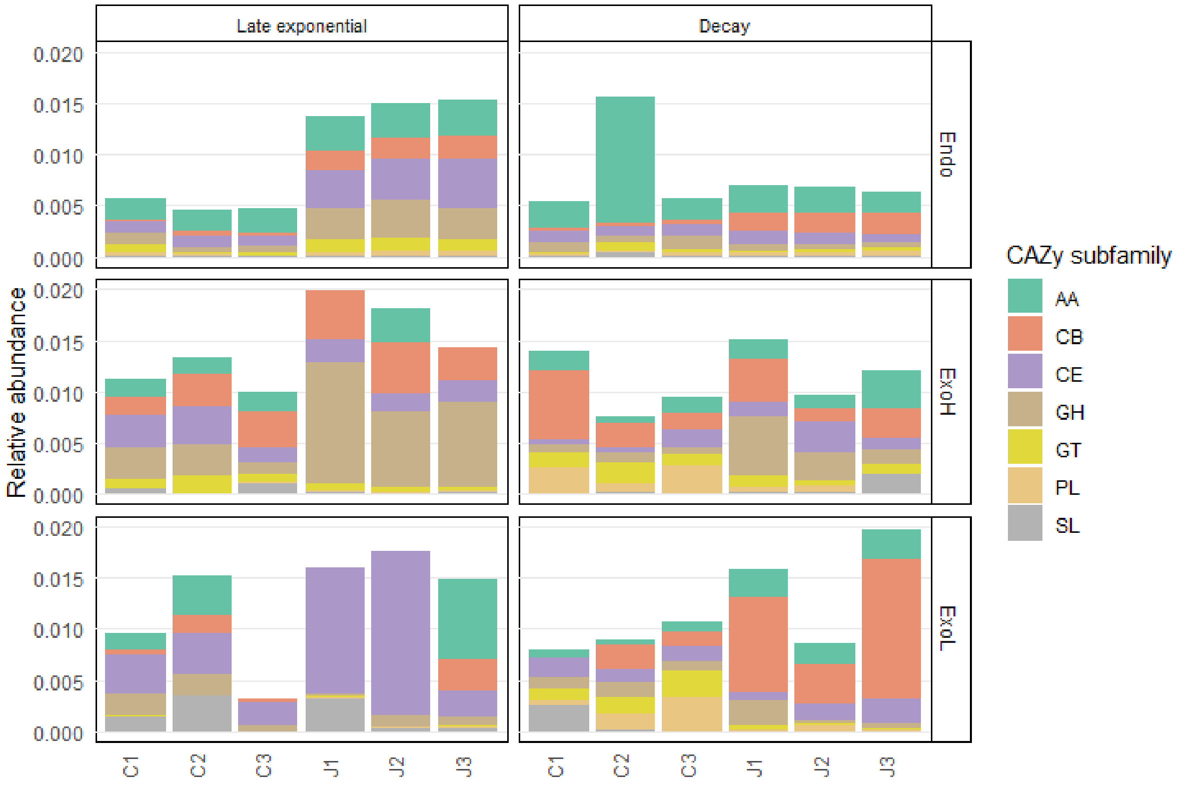

**Figure S3**: Supplementary to Figure 4: High-resolution heatmaps and Venn diagrams of differentially abundant proteins for each key MAG during different stages of the jelly-OM degradation process. **Top panels**: Gene: the total gene profile of each MAG: genes encoding peptidases are in yellow, genes encoding transporters in blue, the rest is shown in grey. The false discovery rate (FDR) of log2 transformed fold change (Log2FC) of all proteins per MAG after jelly-OM amendments during the late exponential (E2_FDR) and decay (E1_FDR) phase, FDR <0.05 is highlighted using (*). Log2FC of individual proteins during late exponential (black dots) and decay phase (red dots) is presented. In the heatmap, jellyfish treatments are highlighted as bars in blue, in orange the control treatments, late exponential phase in dark blue (E2) and decay phase in green (E1). **Bottom panels**: Numbers of differentially abundant proteins for which relative abundance increased (up) or decreased (down) exclusively during late exponential (E2) and/or decay phase (E1) and/or throughout the jelly-OM degradation process are also presented as Venn diagrams.

***Pseudoalteromonas MAG 22***

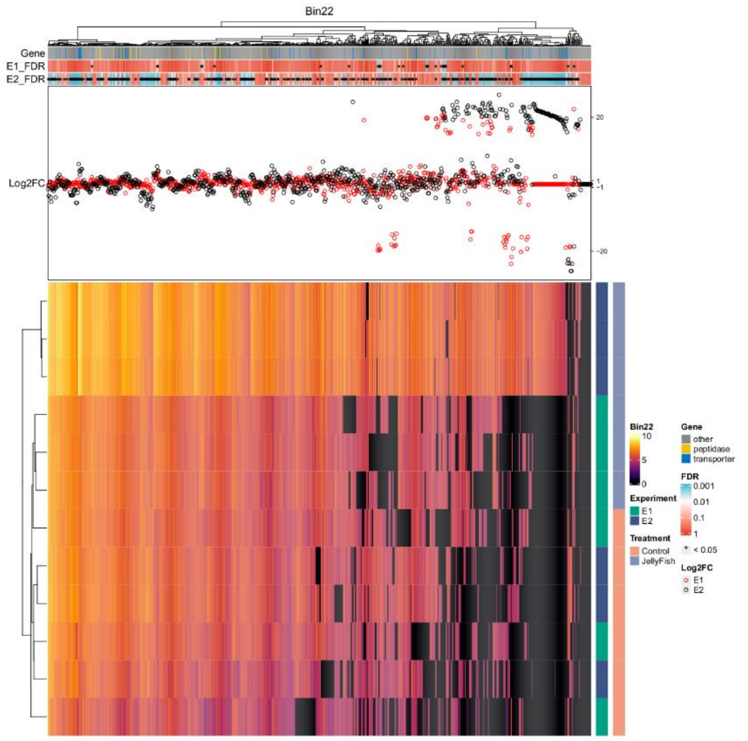

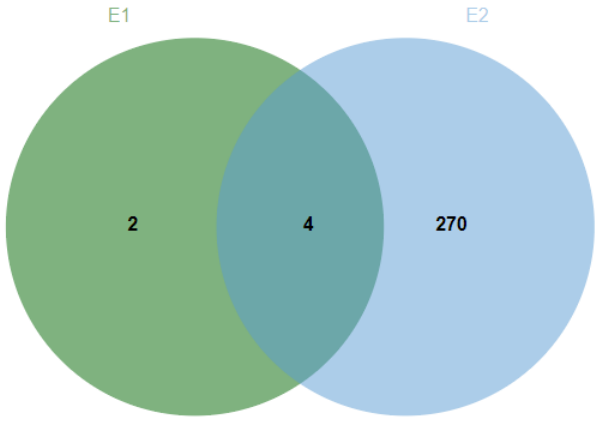
E1
E2
2
4
270

*Pseudoalteromonas MAG 24*

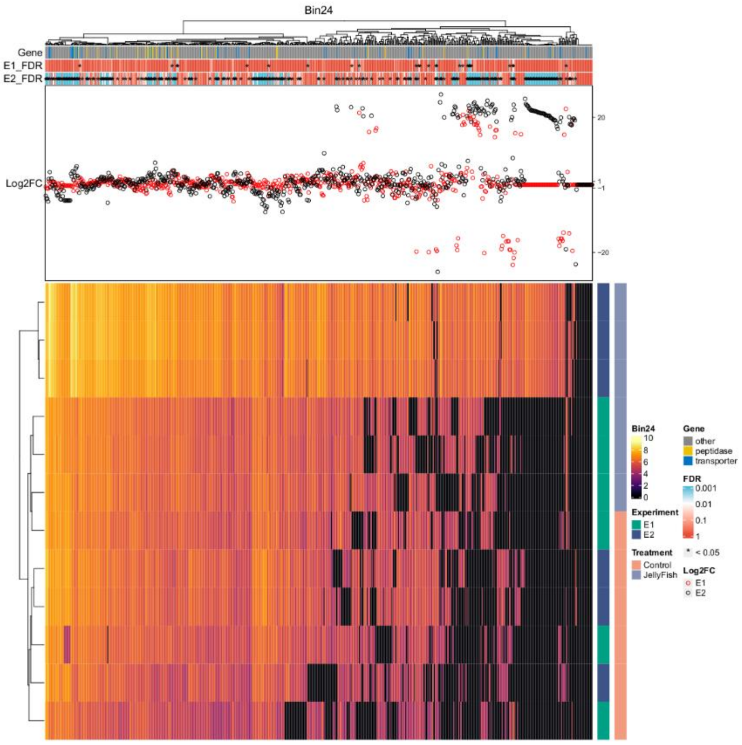


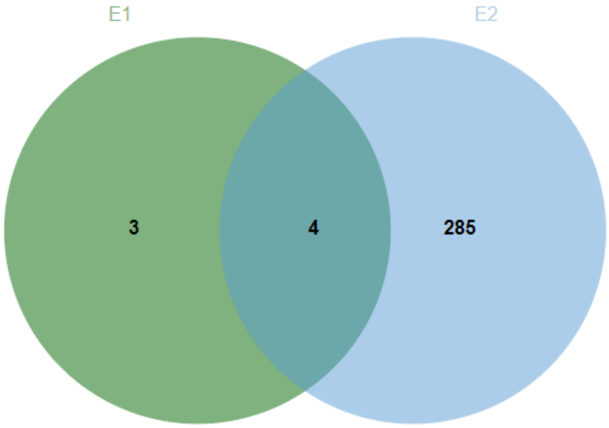

***Vibrio MAG 3***

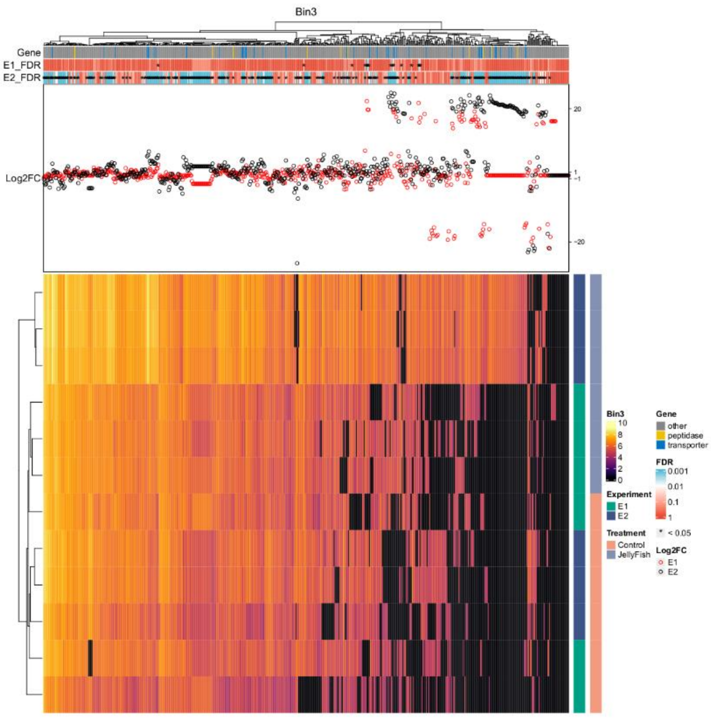
Bin3
Gene
E1_FDR
E2_FDR
Log2FC
20
1
-1
-20
Bin3
10
8
6
4
2
0
Gene
other
peptidase
transporter
FDR
0.001
0.01
0.1
1
* < 0.05
Experiment
E1
E2
Treatment
Control
JellyFish
Log2FC
E1
E2

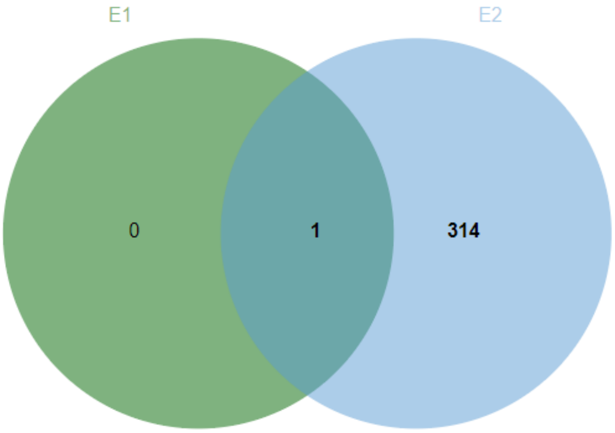
E1
E2
0
1
314

*Alteromonas MAG 29*

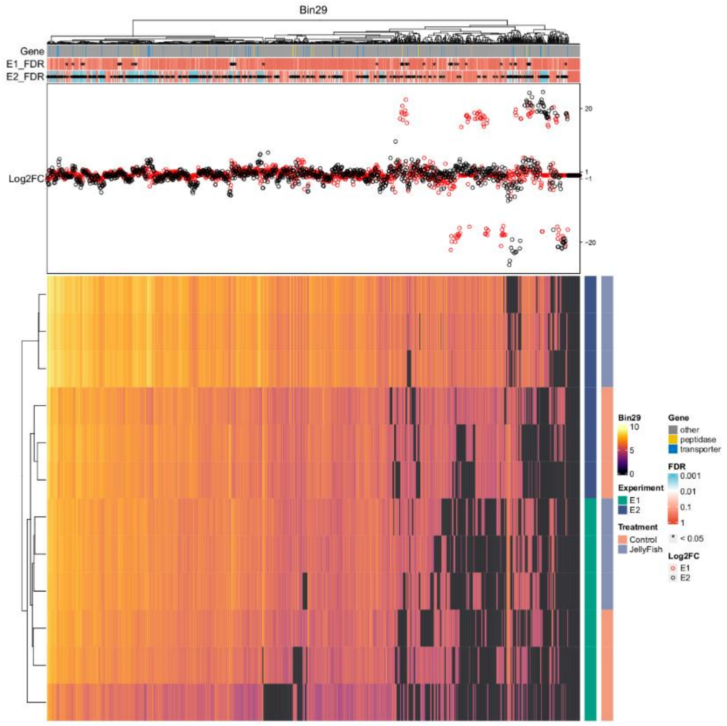


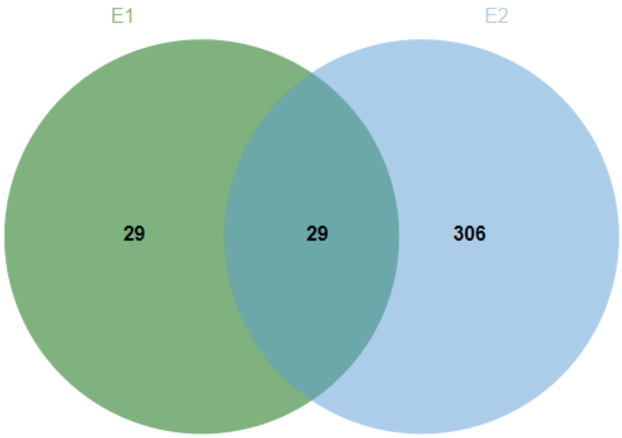

***Thalassobius MAG 51***

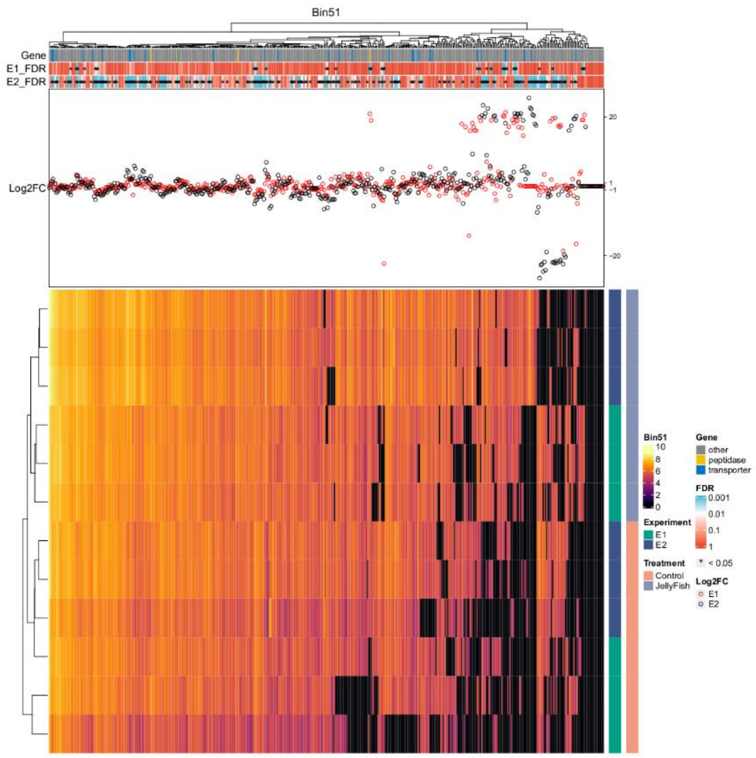


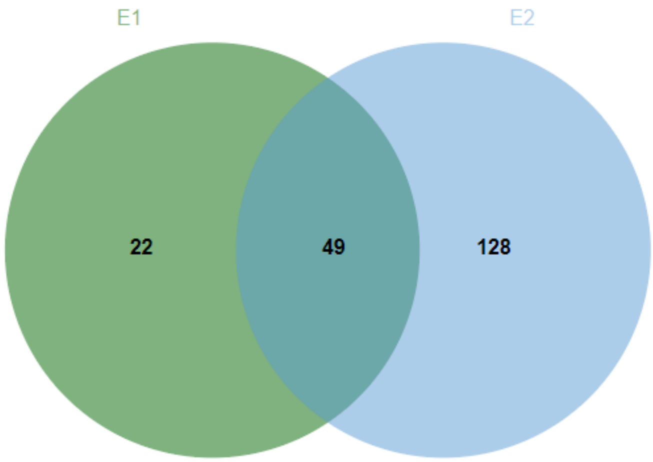

**Figure S4**: **A.**) GO-annotated and **B.**) KEGG-annotated differentially abundant proteins (enrichment as log2FC and significance as p-value or FDR) per each MAG during different stage of jelly-OM degradation process. *Pseudoalteromonas*: bin22 and bin24; *Alteromonas*: bin29; *Vibrio*: bin3; *Thalassobius*: bin51. Circles: proteins related to biological processes, Triangles: proteins related to cellular components, Squares: proteins related to molecular function. Blue: proteins up-regulated in jelly-OM (as compared to control) treatments. Yellow: proteins down-regulated in jelly-OM (as compared to control) treatments. Grey: proteins with no significant difference in expression between jelly-OM and control treatments. E2: late exponential growth phase of jelly-OM degrading community; E1: decay phase of jelly-OM degrading community

**A.**

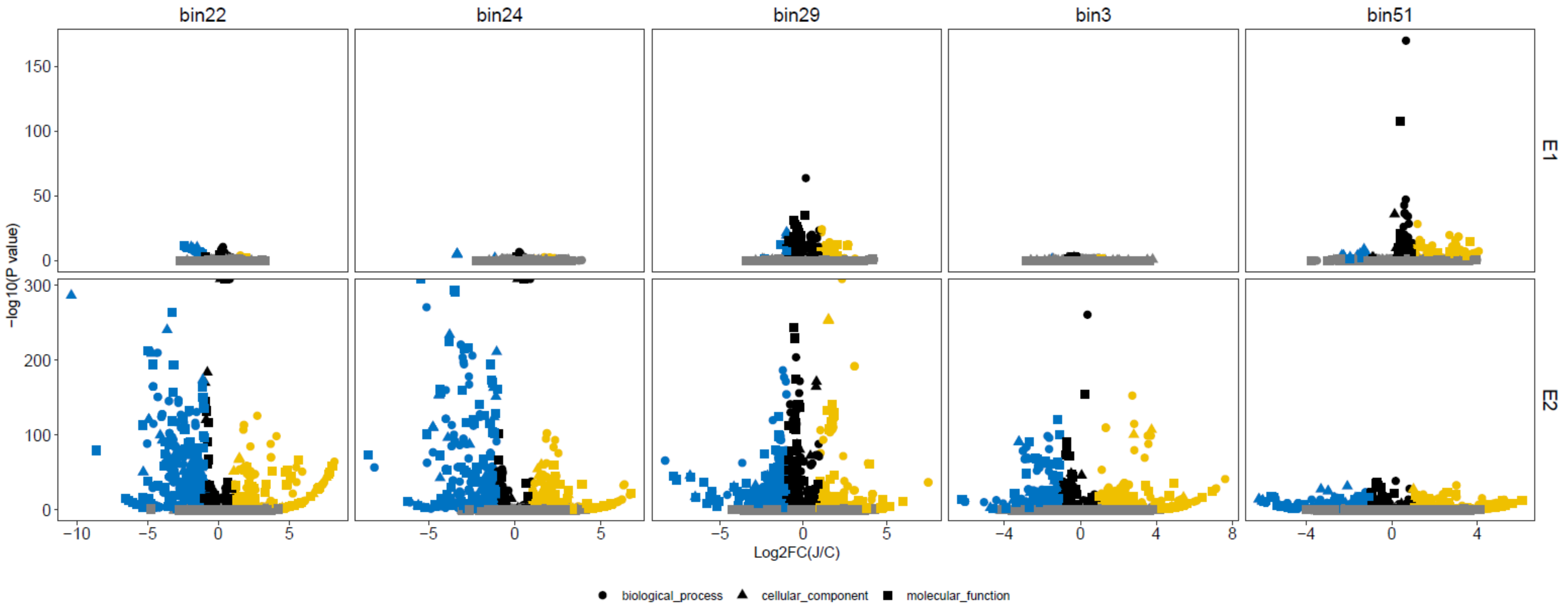

**B.**

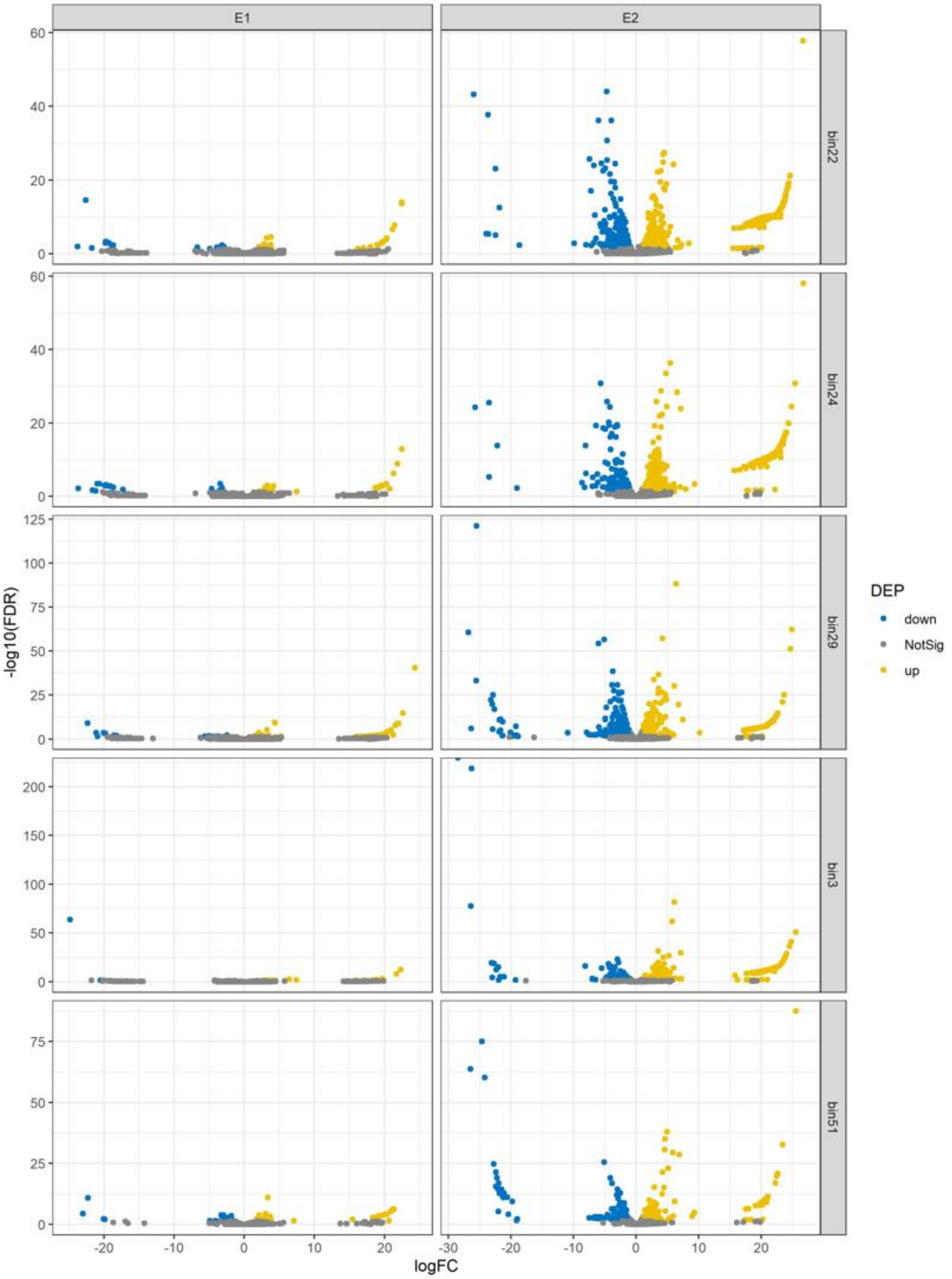
E1
E2
bin22
bin24
bin29
bin3
bin51
-log10(FDR)
logFC
DEP
down
NotSig
up
